# Capturing the Heterogeneity of the PDAC Tumor Microenvironment: Novel Triple Co-Culture Spheroids for Drug Screening and Angiogenic Evaluation

**DOI:** 10.3390/cells14060450

**Published:** 2025-03-18

**Authors:** Ruben Verloy, Angela Privat-Maldonado, Jonas Van Audenaerde, Sophie Rovers, Hannah Zaryouh, Jorrit De Waele, Delphine Quatannens, Dieter Peeters, Geert Roeyen, Christophe Deben, Evelien Smits, Annemie Bogaerts

**Affiliations:** 1 Research Group PLASMANT, Department of Chemistry, University of Antwerp, 2610 Antwerp, Belgium; angela.privatmaldonado@uantwerpen.be (A.P.-M.);; 2 Center for Oncological Research (CORE), Integrated Personalized and Precision Oncology Network (IPPON), University of Antwerp, 2610 Antwerp, Belgium; 3 Department of Pathology, University Hospital Antwerp (UZA), 2650 Antwerp, Belgium; 4 Department of Hepatobiliary Transplantation and Endocrine Surgery, University Hospital Antwerp (UZA), 2650 Antwerp, Belgium

**Keywords:** pancreatic ductal adenocarcinoma, triple co-culture spheroids, tumor microenvironment, drug resistance, heterogeneity, angiogenesis, in vitro 3D model, pancreatic stellate cells, endothelial cells, cancer-associated fibroblasts

## Abstract

Pancreatic ductal adenocarcinoma (PDAC) presents significant treatment challenges due to its desmoplastic reaction, which impedes therapeutic effectiveness, highlighting the need for advanced vitro models to better mimic the complex tumor environment. The current three-dimensional co-culture models of fibroblasts and endothelial cells are lacking, which presents a challenge for performing more comprehensive in vitro research. Our study developed triple co-culture spheroid models using MiaPaCa-2 and BxPC-3 cancer cell lines, with RLT-PSC and hPSC21 pancreatic stellate cell lines and the endothelial cell line HMEC-1. These models were assessed through growth assays, multicolor flow cytometry to optimize cell ratios, cell viability assays to evaluate drug responses, and a tube formation assay with a spheroid-conditioned medium to examine angiogenesis. Our triple co-culture spheroids effectively replicate the PDAC microenvironment, showing significant variations in drug responses influenced by cellular composition, density, and spatial arrangement. The tube formation assay showcased the potential of our models to quantitatively assess a treatment-induced angiogenic response. These cost-effective triple-co-culture in vitro spheroid models provide vital insights into the PDAC microenvironment, significantly improving the quality of the in vitro evaluation of treatment responses.

## 1. Introduction

Pancreatic ductal adenocarcinoma (PDAC) is predicted to become the second leading cause of death by cancer before 2030, clearly emphasizing why improvement in treatment strategy is highly needed [1]. PDAC, which comprises approximately 85% of all pancreatic cancer cases [2], is characterized by a desmoplastic reaction, which triggers the formation of a dense, fibrous tumor stroma causing high intratumoral pressure. This serves as a physical barrier to therapy and causes vascular compression and hypovascularity, which inhibits drug delivery and induces chemoresistance [3]. In addition to their vital role in angiogenesis, endothelial cells (ECs) contribute to the complexity of the disease as cancer-associated fibroblasts (CAFs) through an endothelial-to-mesenchymal transition [4,5]. Furthermore, activated pancreatic stellate cells (PSCs), a specific type of fibroblast, act as the guardians of PDAC and are major contributors to the desmoplasia and the creation of a complex tumor microenvironment (TME). This leads to the acquisition of properties like rapid growth, a highly invasive and metastatic potential; survival in hypoxic and low-nutrient conditions; and immunosuppression, among others [6]. Important point mutations in PDAC are *KRAS*, *SMAD4*, *CDKN2A*, and *TP53* [7]. *KRAS* is detected in 90% of the cases and is related to a worse prognosis alongside *CDKN2A* [8,9]. *SMAD4* is correlated to an increased rate of metastasis and resistance to therapy, and *TP53* changes the immune milieu and promotes inflammation, which benefits the tumor [10,11]. Single-cell RNA sequencing revealed that roughly 35% of all cells in PDAC tumors are cancerous, 26% are fibroblasts and PSCs, and 12% are ECs [12]. While other studies show similar results [13,14,15,16], it is important to note the cellular distribution spread among patients [12,14]. The extreme cellular heterogeneity among patients (intertumoral) and within the tumors (intratumoral) increases the failure rate of therapies in clinical trials and highlights the importance of utilizing more complex models to capture this heterogeneity during the in vitro stage of research [17]. While pharmaceutical research and drug development has been ever-growing, the approval rate of new drugs is not experiencing the same uptrend [18]. This is partially caused by Phase III failures originating from inadequate preclinical screening [19].

To develop novel therapeutic approaches, we need better and more accessible in vitro models that can mimic the TME of PDAC. Simple models such as two-dimensional (2D) cultures have been a useful and economic approach for investigating fundamental questions for decades. However, these models are not sufficient to translate cellular responses to innovative treatment strategies that can tackle these complex diseases [20]. One simple and low-cost alternative is the use of three-dimensional (3D) spheroids, which offer many advantages, including an oxygen gradient, scaffold-free tissue-like cellular organization, cellular crosstalk, high-throughput capabilities, and reproducibility [20,21,22]. Monoculture spheroids of cancer cells lack the stromal component of a tumor-like extracellular matrix (ECM) deposition and a complex TME [21]. It has been stated that co-culture spheroids of a 1:1 and 2:1 ratio (cancer–stellate) are not representative of the PDAC stroma content [22,23,24]. In addition, attempts have been made to develop a triple co-culture (TCC) spheroid model for pancreatic cancer with fibroblasts and endothelial cells. However, to our knowledge, they did not include enough PSCs, which are a highly specific cell population to PDAC that play a pivotal role in the desmoplastic response [25,26]. In these studies, the authors used either lung fibroblasts or extremely low numbers of fibroblasts, which do not truly mimic PDAC tumors, and hence do not provide a full picture of the cell-to-cell interactions governing the PDAC TME. In addition, the intra- and intertumoral heterogeneity of the PDAC TME was not considered.

Another alternative is the use of patient-derived organoids (PDOs), which are gaining attention due to their clinical relevance and their value for understanding patient-specific drug responses for personalized cancer treatment [27]. However, they are reported to have low physical properties and a soft matrix [22,28,29]. Even though PDOs are highly valuable, in many laboratories there is still a lack of expertise and standardized protocols for the processing, culturing, and cryopreservation of these tissues with a high success rate [30]. In addition, most PDOs do not yet include the complex TME related to endothelial cells and angiogenesis, and they require Matrigel or Cultrex (extracellular matrixes isolated from mouse sarcoma) which introduce unknown growth factors [31].

The translatability of in vitro research toward clinical observations will remain low without proper 3D models that consider the complex TME and its heterogeneity with clinically relevant numbers of different cell types to better study the treatment response. In this study, we present a relevant panel of four high-throughput TCC spheroid models of PDAC including both PSCs and ECs. To tackle the heterogeneity of the PDAC TME, we used cell lines with different characteristics for both PDAC cells and PSCs. Altogether, our four novel TCC spheroid models provide an affordable and relevant tool to advance the development of suitable therapies for PDAC that can be widely used for drug screening, angiogenic studies, and more.

## 2. Materials and Methods

### 2.1. Cell Culture

The human pancreatic cancer cell (PCC) lines MiaPaCa-2 (ATCC^®^, Manassas, VA, USA) and BxPC-3 (ATCC^®^) were used in this study. The human immortalized PSC line RLT-PSC was kindly provided by Prof. Ralf Jesenofsky of the Faculty of Medicine, University of Mannheim [32], while the hPSC21 line was established at Tohoku University, Graduate School of Medicine, and was kindly provided by Prof. Atsushi Masamune [33]. In addition, the immortalized endothelial cell line HMEC-1 (ATCC^®^) was used. All cell lines were used for a maximum of 20 passages after thawing and tested for mycoplasma every 3 months, and short tandem repeats (STR) profiling has validated that the cell lines remained identical to the original cell line (Figures S1–S5). Cancer and stellate cells were cultured in Dulbecco’s Modified Eagle Medium (DMEM, 10938025, Gibco, Grand Island, NY, USA) supplemented with 10% foetal bovine serum (FBS, 10270106, Life Technologies, Carlsbad, CA, USA), 100 U/mL penicillin, 100 μg/mL streptomycin (15140122, Life Technologies), and 2 mM L-glutamine (25030024, Life Technologies). Cancer and stellate cells were transduced with NucLight Rapid Red (4741, Essen Biosciences, Ann Arbor, MI, USA) to express mKate2 and NucLight Green (4475, Essen Biosciences) to express GFP respectively. HMEC-1 cells were cultured in MCDB131 (10372019, ThermoFisher Scientific, Waltham, MA, USA) and supplemented with 10 ng/mL Epidermal Growth Factor (PHG0314, ThermoFisher Scientific), 1 µg/mL Hydrocortisone (H0396, Sigma Aldrich, St. Louis, MO, USA), 10 mM L-glutamine (25030024, Life Technologies), 10% FBS (10270106, Life Technologies), 100 U/mL penicillin, and 100 μg/mL streptomycin (15140122, Life Technologies). Cells were maintained in a humidified incubator at 37 °C and 5% CO_2_. HMEC-1 cells for the supplementary proliferation rate were transduced with CMV-GFP.

### 2.2. Proliferation Rate

MiaPaCa-2, BxPC-3, RLT-PSC, hPSC21, and HMEC-1 cells were seeded in a flat 96-well plate (655180, Greiner Bio-One, Kremsmünster, Austria) in 200 µL (1000, 2000, 1000, 1000, and 1500 cells/well, respectively). Cells were either seeded in MCDB131 or DMEM (with or without supplements) to evaluate proliferation over time. Confluence was measured every 24 h using live-cell imaging with the Spark^®^ Cyto (Tecan, Männedorf, Switzerland).

### 2.3. Triple Co-Culture Spheroid Seeding Ratio Optimization

To optimize the seeding densities for triple co-culture spheroids of cancer, stellate, and endothelial cells, initial pilot experiments tested cancer–stellate ratios (1:1 to 1:4) to ensure single, compact spheroid formation and qualitatively assess proportions via imaging (Figure S6a,b). The seeding density was tested in a range from 3000–7500 cells per spheroid to obtain a spheroid of 300–500 µm in diameter. Based on these results, endothelial cells were added to form triple co-cultures, varying cancer–endothelial ratios (1:0.25 to 1:4). Spheroids were analyzed after 3 days using Orbits software (https://www.orbits-oncology.com/) to quantify relative proportions of each cell type (area-based) and were compared to clinical PDAC cellular percentages (Figure S6c,d). Due to the limitations of 2D image analysis, we continued final optimization using flow cytometry to accurately quantify cell-type proportions and refined seeding densities for clinically relevant spheroids.

### 2.4. Spheroid Formation

Three-dimensional co-culture spheroids were generated using the previously mentioned cell lines. Spheroids were seeded at 5000 (BxPC-3:RLT-PSC:HMEC-1 in a ratio of 7:2:4), 7000 (BxPC-3:hPSC21:HMEC-1 in a ratio of 6:5:3), 3500 (MiaPaCa-2:RLT-PSC:HMEC-1 in a ratio of 6:3:3), 4500 (MiaPaCA-2:hPSC21:HMEC-1 in a ratio of 5:6:4), 6000 (BxPC-3), and 4000 (MiaPaCa-2) cells per well, including 0.24% methylcellulose in ultra-low adherent (ULA) plates (7007, Corning^®^, Corning, NY, USA). Empty outer wells were filled with 200 μL of water to minimize evaporation and plates were sealed with a breathable membrane (Z380059, Merck, Rahway, NJ, USA). The ULA plates were centrifuged at 453× *g* for 10 min. Cells were incubated at 37 °C and 5% CO_2_ for three days to promote spheroid formation, after which spheroids of 300–500 μm diameter were formed.

### 2.5. Flow Cytometry

The characterization of the different cell types in the TCC spheroids was performed with multicolor flow cytometry. Forty-eight spheroids per condition were collected after three days of formation and dissociated with 1 mL of TrypLE (12604-021, Life Technologies) for 30 min while shaking and incubating at 37 °C and 5% CO_2_. Mechanical stress was applied by pipetting up and down with 0.1% BSA-coated tips every 10 min. A total of 3 mL of medium was added, cells were pipetted up and down and vortexed, and a single cell suspension was obtained using a 70 µm strainer. All cells were resuspended in 200 µL FACS buffer and seeded in a 96-well round-bottom plate. Subsequently, all cell suspensions were pre-treated with human serum blocking solution (S1-100ML, Merck) for 15 min at room temperature (RT) to avoid non-specific binding and washed twice with FACS buffer. Cells were incubated with PE-Cy7 anti-human CD31 (303118, BioLegend, San Diego, CA, USA) antibody for 15 min at RT, then washed twice with FACS buffer. Cancer and stellate cells were measured with mKate2 and GFP fluorophores that are expressed by the cell nuclei due to transduction, as explained earlier. Samples were measured on the NovoCyte Quanteon (Agilent Technologies, Santa Clara, CA, USA), and analysis of all flow cytometry experiments was performed using the FlowJo v10 software (Becton, Dickinson & Cmopany, Franklin Lakes, NJ, USA).

### 2.6. Cell Viability Assay

Spheroids were formed over three days, as described earlier. The spheroid medium was refreshed by removing 100 µL of medium and adding 100 µL of fresh MCDB131 medium (50% medium renewal). Gemcitabine (GEM, S1714, 10 mM in DMSO, Selleck Chemicals, Houston, TX, USA) and Paclitaxel (PAC, S1150, 10 mM in DMSO, Selleck Chemicals) were applied as monotherapy or as combinational therapy using the D300e Digital Dispenser (Tecan). GEM was titrated using the following concentration range: 2.5 nM, 5 nM, 25 nM, 50 nM, 400 nM, 2.5 µM, and 10 µM with PAC in a 1:5 ratio compared to GEM. Cell viability curves were set up by quantifying ATP from metabolically active cells with CellTiter-Glo^®^ (G7571, Promega, Madison, WI, USA) after five days of drug treatment. DMSO normalization was performed based on the highest drug concentration to a maximum of 0.6% DMSO. Staurosporine (5 µM, S1421, Selleck Chemicals) was used as a 100% cell death control to normalize data. The area under the curve (AUC) value represents the area under the curve and is a measure for drug resistance. The higher the viability, the larger the AUC value will be, therefore indicating higher drug resistance.

### 2.7. Enzyme-Linked Immunosorbent Assay

Supernatant levels of VEGF in triple co-culture spheroid-conditioned media were quantified using the LEGEND MAX™ Human VEGF ELISA Kit (446507, BioLegend) according to the manufacturer’s instructions. Absorbance was measured at 450 nm and 570 nm (reference) using the Spark^®^ Cyto (Tecan).

### 2.8. Tube Formation Assay

Assessment of angiogenesis was performed with a tube formation assay in a 384-well plate (3764, Corning^®^). The supernatant (spheroid-conditioned medium) of 3-day-old TCC spheroids cultured in 200 µL was refreshed by either renewing 100 µL with fresh medium to a total of 200 µL or removing 150 µL of medium and adding 50 µL of fresh medium for a total of 100 µL, both leading to a 50% medium refresh (indicated as 100 µL or 200 µL in Figure 4a). Plates were then coated with 8 µL of Cultrex (3533, Bio-Techne, Minneapolis, MN, USA), centrifuged at 180× *g* for three min and incubated for at least 60 min at 37 °C and 5% CO_2_. HMEC-1 cells were resuspended at 58,000 cells/mL (either in MCDB131 or spheroid-conditioned medium), and 40 µL of cell suspension was seeded on top of the Cultrex coating. Fresh MCDB131 medium was used as a positive control, as this highly supports tube formation and loops. After 6 h, images of the tubular network were taken using the Spark^®^ Cyto (Tecan) with a 4× objective. This protocol was adapted to our application based on the literature [34,35]. Python v3.12 was used to crop these images to a center-focused circular image to remove the out-of-focus outside regions of the well, caused by the meniscus shape of the Cultrex coating. Finally, the IKOSA^®^ tube formation analysis (Kolaido, Altenrheim, Switzerland) was performed to gain four metrics: number of loops, covered area, total tube length, and number of branch points.

### 2.9. Patient Samples

FFPE (Formalin-fixed paraffin-embedded) tissue blocks from 10 patients with pancreatic ductal adenocarcinoma (PDAC) were acquired from the Antwerp Biobank (Antwerp, Belgium; ID: BE71030031000). The usage of these samples was approved by the Ethics Committee at Antwerp University Hospital–University of Antwerp (UZA–UAntwerp) under the reference number EC14/47/48. Two patient samples were selected as representative tumors to represent multiple typical PDAC characteristics.

### 2.10. Immunohistochemistry

Alpha-smooth muscle actin (α-SMA) staining was part of a previous study [36]. ETS-related gene (ERG) expression in the TME of clinical PDAC tumors was evaluated by staining for hematoxylin and eosin (H&E) and ERG of five µm-thick sections from formalin-fixed paraffin-embedded tissue blocks. Sections were baked at 60 °C for 2 h and heat-induced epitope retrieval was performed by incubation with Envision FLEX high pH antigen retrieval solution (GV80411-2, Dako, Glostrup, Denmark) for 20 min at 97 °C (PT-Link instrument, Dako). Next, peroxidase activity was quenched by incubating the slides in peroxidase blocking buffer (GE001, Dako) for 5 min. Incubation with an anti-ERG monoclonal antibody (GA65961-2, monoclonal anti-rabbit, clone EP111, Agilent Technologies) was performed for 20 min at room temperature. Visualization was achieved by using a rabbit-linker (K800921-2, Agilent) for 10 min and the Envision FLEX detection kit (K802321-2, Agilent) for 20 min according to the manufacturer’s instructions. All sections were counterstained with hematoxylin (105175, Merck) for 2 min, dehydrated, and mounted with mounting medium (SEA-1604-00A, Cellpath, Wales). Images were captured from digitized histological slides by using a Philips Ultra-Fast scanner and displayed at 200× final magnification.

### 2.11. Spheroid Image Analysis

Images of the TCC spheroids were captured using the non-confocal Spark^®^ Cyto (Tecan) microscope with a 10× objective. Image acquisition was performed for brightfield, green (stellate), red (cancer), and blue (endothelial) fluorescent channels (Table 1).

### 2.12. Statistical Analysis

All experiments were performed at least three times with three replicates unless specified otherwise. The significances in the proliferation assay were evaluated using two-way ANOVA with Sidak’s multiple comparison test using Prism v10.1.0 (GraphPad Software, San Diego, CA, USA). Dose-response curves were evaluated for significances using the AUC values (*p* ≤ 0.05) and a linear mixed model with either the treatment or the spheroid combination as fixed effect using JMP Pro v17.0.0 software. Outlier tests were performed using Prism v10.1.0 (GraphPad Software). For multiple comparisons of all experimental groups, Tukey’s honestly significant difference (HSD) was used. Statistical significances between the concentrations of VEGF in spheroid-conditioned media was also evaluated with linear mixed models using JMP Pro v17.0.0 software (JMP, Cary, NC, USA) and Tukey’s HSD multiple comparisons test.

## 3. Results

### 3.1. MCDB131 Medium Is Most Optimal for Triple Co-Culture Spheroid Formation

To include the heterogeneity of the PDAC TME, we used two PCC lines with different genetic backgrounds and associated characteristics (MiaPaCa-2 with *CDKN2A* gene deletion and *KRAS* and *TP53* mutations, BxPC-3 with *CDKN2A* and *SMAD4* gene deletion and *BRAF*, and *TP53* mutations). PANC-1 and Capan-2 were also assessed in a preliminary monoculture spheroid formation assay. Unlike MiaPaCa-2, PANC-1, and Capan-2, BxPC-3 formed compact spheroids and was selected to introduce structural heterogeneity in our spheroid models. Capan-2 did not even form loose singular spheroids like MiaPaCa-2 and PANC-1. We decided to continue with MiaPaCA-2, which is frequently used in our lab and widely reported in the literature. Additionally, to increase heterogeneity, we also used two PSC lines with different origins (RLT-PSC originating from chronic pancreatitis, as this is one of the main causes of pancreatic cancer development, and hPSC21 originating from pancreatic cancer) and one endothelial cell line (HMEC-1) [32,33]. As PCCs, PSCs, and ECs were cultured in different media, we evaluated the cell growth of each cell line in both DMEM (PCC and PSC culture medium) and MCDB131 (EC culture medium) to determine the optimal medium for TCC spheroid formation. Clearly, MCDB131 was able to sustain growth for all cell lines, similar to DMEM (MiaPaCa-2 and RLT-PSC) or better than DMEM (BxPC-3, hPSC21, and HMEC-1) (Figure S7). The DMEM medium supplemented with VEGF and FGF was not able to provide the required environment for ECs to grow to the same extent as they did in the MCDB131 medium (Figure S8). Therefore, the MCDB131 medium was chosen for spheroid culture.

### 3.2. Triple Co-Culture Spheroids Demonstrate the Heterogeneity of the PDAC Tumor Microenvironment

**Figure 1:**
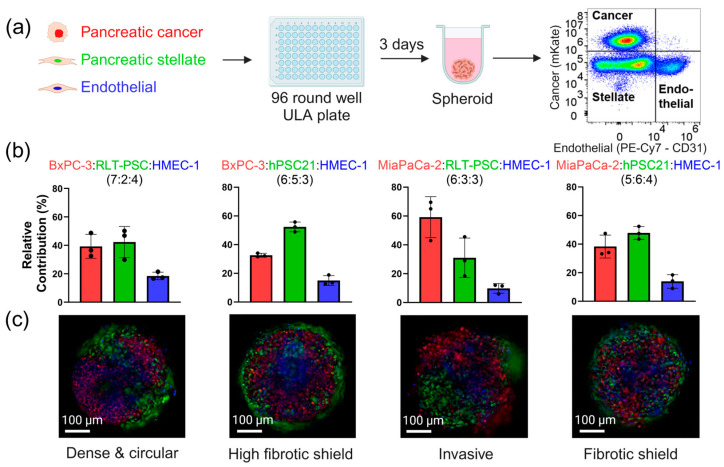
Capturing the heterogeneity of PDAC in the scope of the TME with TCC spheroids. (**a**) Methodology for spheroid formation of pancreatic cancer (red fluorescently labelled nuclei), stellate (green fluorescently labelled nuclei), and endothelial cells (CD31 positive). (**b**) Quantitative flow cytometric data showing the relative contribution of each cell population in our four spheroid combinations. (**c**) Representative live images of the TCC spheroids of pancreatic cancer (red), stellate (green), and endothelial (blue) cells. Data are represented as mean ± SD. Each dot represents one flow-cytometry measurement of a pool of 48 spheroids. ULA: ultra-low attachment.

To generate TCC spheroids with different characteristics of PDAC tumors, we first determined the optimal seeding densities for each cell type in a TCC environment. For this, we quantified the cell populations in 3-day-old spheroids by flow cytometry (Figure 1a and Figure S9). The seeding densities for each TCC spheroid type was adjusted to achieve similar population percentages in 3-day-old spheroids to those observed in patients’ tumors, as reported in the literature [12,13,14,15,16]. These adjustments were made based on the quantitative data from the flow cytometry experiments (Figure S10). The final seeding ratios of the four distinct combinations of TCC spheroids that presented characteristics of clinical tumors and the different facets of the heterogeneous spectrum of PDAC (Figure 1b) were as follows: 7:2:4 BxPC-3:RLT-PSC:HMEC-1 (dense and round), 6:5:3 BxPC-3:RLT-PSC:HMEC-1 (well-defined fibrotic shield), 6:3:3 MiaPaCa-2:RLT-PSC:HMEC-1 (invasive PCCs protruding from spheroids), and 5:6:4 MiaPaCa-2:hPSC21:HMEC-1 (less-dense fibrotic shield). All four TCC combinations were able to form compact spheroids with diameters of 300–500 µm at 3 days post-seeding (Figure 1c). Notably, the seeding ratios of the different cell types did not correlate fully with the flow cytometry ratios after 3 days of formation (Figure 1b). This shows that the growth of these cells in a 3D co-culture environment is different from their 2D monoculture growth (Supplementary Figure S7).

We observed that the stellate cell line hPSC21 (which originates from pancreatic cancer) tended to organize itself around the tumor (Figure 1c), shielding it from the environment, a characteristic often seen in clinical PDAC tumors (Figure S11) and which has been linked to drug resistance. When this shield was not present (as observed in the spheroids generated with RLT-PSC that originates from chronic pancreatitis), the highly invasive nature of MiaPaCa-2 lead to the migration of PCCs out of the TCC spheroids, something not observed in TCC spheroids generated with BxPC-3. The TCC spheroid models showed typical characteristics of PDAC, including the presence of abundant fibroblasts with a fibrotic shield in some cases, invasive tumor edges (as observed in MiaPaCa-2 spheroids, Figure 1c), and a low number of endothelial cells (characteristic of the limited vasculature present in PDAC tumors), as observed in human PDAC samples (Figure S11).

Altogether, these results show that the four different TCC spheroids developed here resemble PDAC tumor characteristics and have significant potential to improve in vitro research.

### 3.3. Improved Spheroid Compactness in the Triple Co-Culture Microenvironment Better Mimics the Solid Nature of PDAC

**Figure 2:**
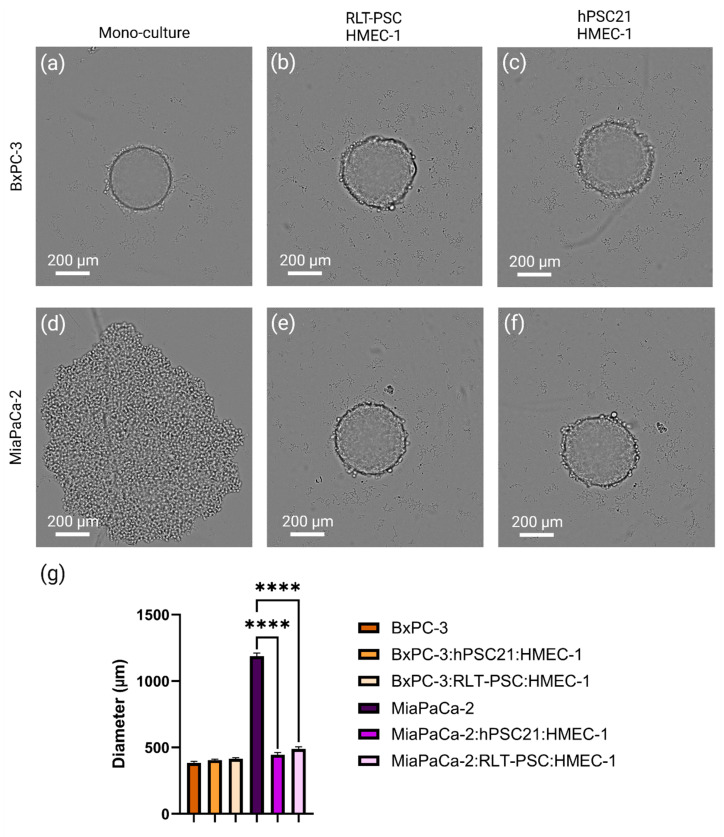
A triple co-culture environment ensures a dense spheroid structure. Representative brightfield images of (**a**) BxPC-3, (**b**) BxPC-3:RLT-PSC:HMEC-1 (7:2:4), (**c**) BxPC-3:hPSC21:HMEC-1 (6:5:3), (**d**) MiaPaCa-2, (**e**) MiaPaCa-2:RLT-PSC:HMEC-1 (6:3:3), and (**f**) MiaPaCa-2:hPSC21:HMEC-1 (5:6:4) spheroids. (**g**) Quantitative evaluation of the spheroid diameter as a mean of the width and length. Data are represented as mean ± SD (n = 9 from three independent experiments for TCC spheroids and n = 3 from one experiment for monoculture spheroids). **** = *p* ≤ 0.0001.

Given the solid nature of PDAC tumors, we quantitatively evaluated the spheroid diameter and qualitatively evaluated the compactness and general spheroid structure using live cell imaging (Figure 2), which we compared for TCC versus monoculture spheroids. We observed that both the monoculture and TCC spheroids of BxPC-3 cancer cells formed compact spheroids (Figure 2). In contrast, MiaPaCa-2 monoculture formed loose cell aggregates of roughly 1000 µm in diameter, which is different from the more solid and tightly packed structure observed when MiaPaCa-2 was seeded in combination with PSCs and endothelial cells (approx. 400 µm diameter; Figure 2). This ability of MiaPaCa-2 to form compact spheroids upon co-culturing with PSCs and ECs highlights the influence and importance of the TME on the structure of PDAC tumors. This improved compactness in TCC spheroids is relevant, as PDAC is known to form highly solid and dense tumors in patients.

### 3.4. Validation of Four Spheroid Models Incorporating Tumor Microenvironment Heterogeneity for Drug Screening

**Figure 3:**
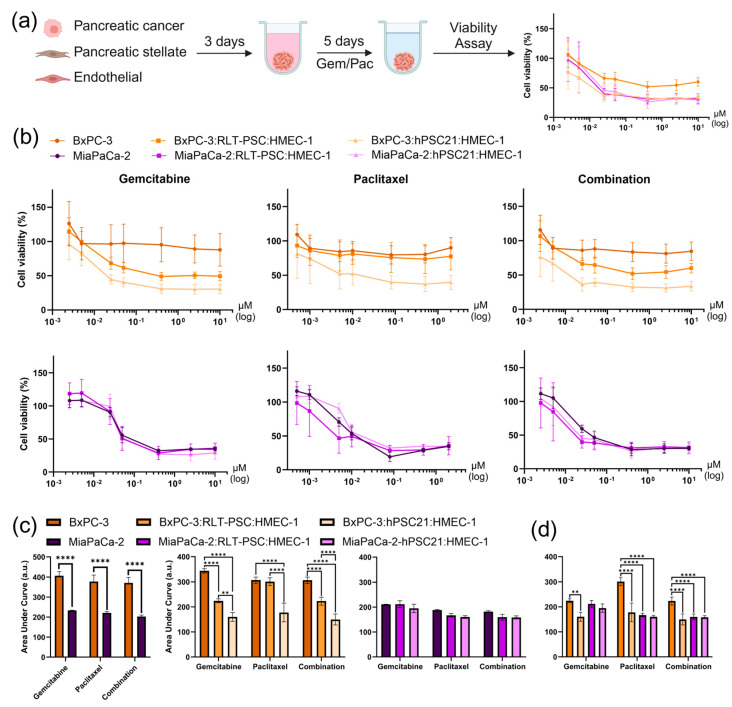
Gemcitabine and paclitaxel treatments of monoculture and TCC spheroids reveal different drug resistance profiles depending on the tumor microenvironment. (**a**) Methodology scheme to measure the cell viability of spheroids with CellTiter-Glo^®^ after 5 days of drug treatment. (**b**) Cell viability curves of gemcitabine- and paclitaxel-treated monoculture and TCC spheroids. The *x*-axis in the combination plots corresponds to the gemcitabine concentrations used; however paclitaxel was co-administered in a 1:8 ratio (paclitaxel–gemcitabine). (**c**) Area under the curve as a measure of drug resistance of monoculture alone, and a comparison of monoculture and TCC spheroids per cancer cell line. (**d**) Area under the curve results of TCC spheroids. Data are represented as mean ± SD (n ≥ 7 from three independent experiments). All data were normalized to an untreated and 100% cell death control. Gem: gemcitabine; Pac: paclitaxel; A.u.: arbitrary units. Statistics were performed using linear mixed models with either the treatment or the spheroid combination as fixed effect using JMP Pro v17.0.0 software. For multiple comparisons of all experimental groups, Tukey’s honestly significant difference (HSD) was used. ** = *p* ≤ 0.01; **** = *p* ≤ 0.0001.

To determine the value of the four distinct TCC spheroid models in drug response studies, we investigated the effect of GEM and PAC as single and combination treatments. Monoculture and TCC spheroids were treated for 5 days, and the area under the cell viability curve AUC was used as a metric for drug resistance, as some viability curves did not drop below 50% viability, which made IC50 calculation impossible (Figure 3a,b).

Contrasting drug resistance was observed in the monoculture PCC spheroids. BxPC-3 monoculture spheroids displayed higher drug resistance than the MiaPaCa-2 spheroids (Figure 3b,c). This could be explained by the structural differences (Figure 2) and different intrinsic characteristics due to the genetics of both cell lines. Remarkably, the BxPC-3 monoculture spheroids exhibited higher drug resistance than BxPC-3 TCC spheroids (Figure 3b,c). In contrast, the presence of other stromal cells in the TCC spheroids containing MiaPaCa-2 did not affect the spheroid’s sensitivity to the treatments, indicating a lower influence of the triple co-culture TME in the case of the MiaPaCa-2 spheroids (Figure 3b,c).

TCC spheroids generated with RLT-PSC in combination with BxPC3 or MiaPaCa-2 showed differences in their sensitivities to GEM, PAC, or both (Figure 3d). However, these differences were not present in TCC spheroids generated with hPSC21. Interestingly, BxPC-3:RLT-PSC:HMEC-1 spheroids exhibited higher resistance than any of the other three TCC spheroid models, as seen from the AUC values (Figure 3d). This suggests a distinct resistance profile in the context of the complex TME represented by this specific TCC spheroid.

Altogether, our results demonstrate that the different TMEs in our four spheroid combinations affect the drug response differently and highlight the importance of considering the TME heterogeneity in drug response studies.

### 3.5. Triple Co-Culture Spheroids and Angiogenesis: Exploring Treatment Response

**Figure 4:**
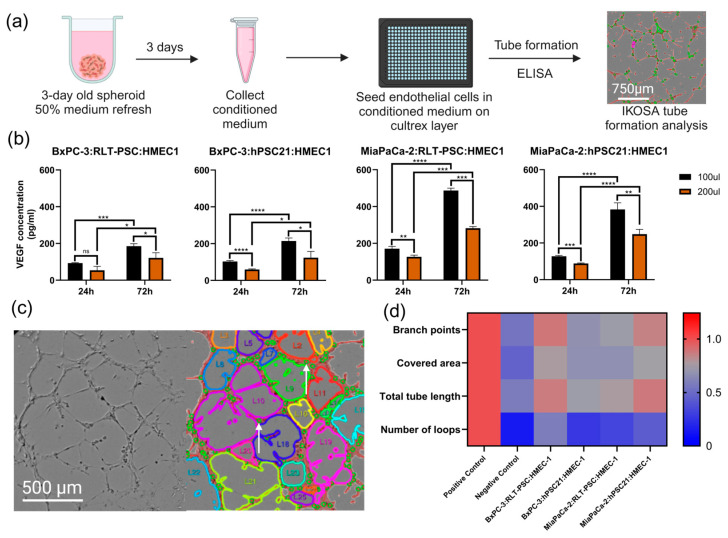
Heterogenous PDAC TCC spheroids display distinct angiogenic profiles. (**a**) Methodology scheme of using spheroid-conditioned medium for a tube formation assay to evaluate angiogenesis. Three-day old spheroids could be treated with an angiogenic treatment schedule right after medium refreshment. (**b**) VEGF concentrations of spheroid-conditioned medium after 24 h or 72 h by ELISA (n = 3 from one independent experiment). (**c**) Raw (left) and IKOSA^®^ analysis images (right) of a positive control showing tubes (red lines), branch points (small green circles, white arrow), and a high number of loops (colored perimeter lines). (**d**) Heat map of the IKOSA^®^ tube formation analysis results for the positive and negative controls and the four combinations of untreated TCC spheroid-conditioned media (n ≥ 8 from three independent experiments). Data are represented as mean ± SD. Statistics were performed using linear mixed models with either the treatment or the spheroid combination as the fixed effect and using JMP Pro v17.0.0 software. For multiple comparisons of all experimental groups, Tukey’s honestly significant difference (HSD) was used. * = *p* ≤ 0.05; ** = *p* ≤ 0.01; *** = *p* ≤ 0.001; **** = *p* ≤ 0.0001; ns = not significant.

Lastly, we investigated the value of our TCC spheroid models in angiogenesis studies using the tube formation assay in vitro (Figure 4a). We evaluated the presence of VEGF in the spheroid-conditioned medium when spheroids were cultured in 100 or 200 µL and after 24 or 72 h post-seeding. We concluded that a medium renewal of up to 100 µL 72 h post-seeding provided the highest VEGF concentrations (Figure 4b). In addition, we observed that MiaPaCa-2 TCC spheroid-conditioned media presented higher levels of VEGF than spheroid-conditioned media from BxPC3 TCC spheroids, showing a two-fold increase in TCC spheroids with hPSC21 (BxPC-3:hPSC21:HMEC-1, 214 pg/mL; MiaPaCa-2:hPSC21:HMEC-1; 383 pg/mL; *p* ≤ 0.0001) and a three-fold increase in TCC spheroids with RLT-PSC (BxPC-3:RLT-PSC:HMEC-1, 185 pg/mL; MiaPaCa-2:RLT-PSC:HMEC-1; 487 pg/mL; *p* ≤ 0.0001).

Tube formation in the endothelial cell monolayers was evaluated using the IKOSA^®^ tube formation analysis, which provides four key parameters: number of loops, total tube length of the vessel network, total covered area of the vessels, and number of branch points (Figure 4c,d). In combination with the number of loops, the total tube length and covered area offered a better understanding of angiogenesis than the number of tubes alone, as the negative control often gave a high number of tubes due to the presence of single cells and disconnected short tubes. However, the presence of loops (Figure 4c,d positive control) represents a mature network of tubes [37], which was not present in the negative control or the spheroid-conditioned media. This correlates to the vascular compression and hypovascularity of PDAC.

Compared to our negative and positive controls, BxPC-3:RLT-PSC:HMEC-1 and MiaPaCa-2:hPSC21:HMEC-1 spheroid-conditioned media showed high numbers of branching points, covered areas, and total tube lengths. In contrast, BxPC-3:hPSC21:HMEC-1 and MiaPaCa-2:RLT-PSC:HMEC-1 spheroid-conditioned media induced a lower number of branch points, total tube lengths, and covered areas (Figure 4d), Additionally, the number of loops is an interesting metric for studying a proangiogenic response, as none of the untreated spheroid-conditioned media resulted in a high number of loops, similar to the negative control (Figure 4d). Our results demonstrate that the four TCC spheroids are valuable models for evaluating pro- or anti-angiogenic treatments, as shown by the differential production of VEGF in the spheroid-conditioned medium and the angiogenic response evoked in the endothelial cells.

Overall, we developed four spheroid models that mimic the TME heterogeneity and its unique characteristics observed in patients with valuable potential for low-throughput screening of drug treatment and angiogenic evaluation (Table 2).

## 4. Discussion

The complex and heterogeneous TME of PDAC, which is characterized by a dense stroma, limits drug delivery and can cause chemoresistance [3]. Recognizing the crosstalk between different cell types is essential for tackling PDAC and developing better screening tools for drug discovery [4,5,6]. In this study, we developed an innovative panel of four TCC spheroid models that represent the complex TME observed in different types of PDAC tumors, with low-throughput secondary screening potential for anti-cancer drugs and angiogenic compounds. Traditional 2D and monoculture models struggle with translatability towards in vivo and clinical studies, while the adoption of 3D co-culture in vitro models, particularly our clinically relevant and heterogeneous TCC spheroids, signify an important shift in PDAC research [20,21,22].

To generate the four TCC spheroid models presented in this study, we considered the literature-derived cell percentages observed in clinical tumors, determined by single-cell RNA sequencing (approximately 35% cancer cells, 26% fibroblasts and PSCs, and 12% ECs, among other cell types with a notable spread between patients) [12,14], which is also described in other studies [13,14,15,16]. To account for the heterogeneity of the TME, a hallmark of PDAC, we used two different PCC and PSC lines, mirroring the inter- and intratumoral complexity and diversity, as well as cellular distribution, as observed in patient tumors [22]. Two TCC spheroid models containing hPSC21 cells presented different levels of fibrotic shields that correlate with the presence of desmoplastic tissue in PDAC samples. In contrast, TCC spheroids containing RLT-PSC cells formed dense and round spheroids (TCCs with BxPC3), as observed in the tightly packed invasive front in PDAC tumors, or a more disperse distribution, characteristic of more aggressive and migratory PDAC (TCCs with MiaPaCa-2). It is known that BxPC-3 cells form dense spheroids that mimic the compactness of PDAC, and this could result in poor drug penetration and high drug resistance [38]. In contrast, the structural support provided by PSCs and ECs to the TCC spheroids prevented the formation of loose spheroids with the aggressive MiaPaCa-2 cell line. This improved their compactness, which is true of clinical PDAC tumors, and resistance to common laboratory manipulation, e.g., pipetting of the spheroids without breaking their structure. This is an advantage of our TCC spheroids, because they allow the MiaPaCa-2 cell line to be investigated in a relevant 3D in vitro model, which is not possible otherwise with the overly loose and fragile monoculture cell aggregates.

It is important to consider the fact that MiaPaCa-2 presents more aggressive features than BxPC-3, such as a high expression of the mesenchymal marker vimentin, important for migration and invasion, and low expression of the epithelial marker e-cadherin, a tumor-suppressor protein involved both in epithelial-mesenchymal transition and tumor progression [39]. Indeed, it has been demonstrated that BxPC-3 spheroids express higher levels of cadherins than MiaPaCa-2, which are essential for spheroid formation [40]. Interestingly, we also observed the formation of a cluster of endothelial cells in the core of 3-day old BxPC-3:hPSC21:HMEC-1 spheroids. This is in agreement with the distribution of ECs observed in 4-day-old PANC-1:MRC-5:HUVEC spheroids, where the cluster of HUVECs present in the core was progressively lost after 7 days [25]. Thus, it is possible that the initial aggregation of ECs observed here will not lead to the formation of capillary-like structures and will result in hypovascularized 3D spheroids. The structural differences observed between monoculture and TCC spheroids highlight the importance of considering spatial arrangement, cell density, and the TME for mimicking clinical scenarios. The lack of crosstalk among different cell types in monoculture spheroids impacts various aspects, including responses to immunotherapy, growth rates, survival, invasion, metastasis, angiogenesis, and other critical factors [22,41].

To validate the use of our models in drug screening, we assessed the drug response to first-line chemotherapeutics GEM and PAC in both monoculture and TCC spheroids. In our study, BxPC-3 monoculture spheroids exhibited higher resistance to treatment than BxPC-3 TCC spheroids. This agrees with a previous study demonstrating that BxPC-3 3D spheroids were more resistant to GEM than 2D cultures, although they only used monoculture 3D spheroids [42]. In addition, in our study, the BxPC-3 spheroids demonstrated higher resistance to chemotherapy than the MiaPaCa-2 spheroids, and this was partially reversed in BxPC-3 TCC spheroids. Conversely, the TME of the TCC spheroids containing the MiaPaCa-2 cells did not significantly affect the overall survival of the cells exposed to chemotherapy. However, there are contradictory reports regarding the sensitivity to chemotherapeutic drugs of these PDAC cell lines. While BxPC-3 has been reported to be more sensitive to GEM than MiaPaCa-2 in 2D cultures [43,44], another study reported the opposite [45]. This discrepancy could reflect the different responses achieved in the presence of a more complex TME and validates the use of diverse cell lines and TMEs exhibiting varying sensitivity to chemotherapy. This allows for a more comprehensive understanding of the dynamics of drug resistance. The differential drug response observed in TCC spheroids suggests specific interactions between cancer and stromal cells. Indeed, the genotypic and phenotypic characteristics of each cell line contribute to the creation of unique TMEs that influence growth, angiogenesis, and drug resistance, among others, where the specific communication between cancer cells and PSCs is a key component [46]. The observed difference in drug responses in our TCC spheroid models matched the patient-specific drug resistance profiles recently shown in PDOs [47]. In this regard, we propose to use at least two TCC spheroid combinations to represent a highly resistant tumor (BxPC-3:RLT-PSC:HMEC-1) and a drug-sensitive tumor (MiaPaCa-2:hPSC21:HMEC-1). Both TCC spheroids consist of different PCC and PSC lines, which ensures heterogeneity. Our drug regimen serves as a proof-of-concept for TCC spheroid models and can be expanded to a broader spectrum of therapeutic agents for future investigations and high-throughput drug screening, assessing cell death of each cell type within the TCC spheroids to determine the effects of chemotherapy in a TCC environment.

We also tested the ability of our TCC spheroid models to induce angiogenesis, a hallmark of cancer, as the vasculature plays an important role in drug response, invasion, metastasis, and tumor growth [48]. It is known that cancer cells can release VEGF and other proangiogenic factors that modulate the development of endothelial cells [49]. Using a tube formation assay and a spheroid-conditioned medium, we observed that TCC spheroids containing the same type of cancer cells produced similar levels of VEGF; however, the angiogenic potentials of the spheroid-conditioned media of each of them were different. These results suggest the presence of other angiogenic factors, such as TNF-α, IL-1β, or IL-6, produced by the different cells of the TCCs [50]. A limitation of our model in this regard is the use of the commercial cell line HMEC-1, which is a dermal microvascular endothelial cell line that is not from pancreatic origin. Further research is needed to determine the types of factors and the cells in the TCCs secreting them that are responsible for the induction or inhibition of angiogenesis.

In comparison to the state-of-the-art 3D co-culture models [25,26], we were able to include CAFs, more specifically PSCs, in a clinically relevant ratio to tumor cells, which created cell-to-cell interactions that reflected the complex TME of PDAC, as demonstrated by the comparison with the patient samples. Unlike other triple co-culture spheroid models [25,26], we developed four spheroid models that recreated the spread of cellular distribution observed among the patients and the TME heterogeneity, each of them with unique characteristics (Table 2). Our TCC spheroid models overcome some of the challenges still faced by PDOs, as the TCC spheroid models presented here incorporate the complex TME of PDAC while remaining a simple, low-cost, and easily accessible model suitable for high-throughput screening. Moreover, our TCC model can be combined and extended with PDOs, following full characterization and biobanking. We believe our models can also incorporate immune cells to provide an even more comprehensive understanding of the role of TME in treatment response, particularly in the context of immunotherapy [51]. Additionally, our triple co-culture models can easily be extended toward PDOs instead of pancreatic cancer cell lines, which would improve the clinical translatability of our models even further. Combined, our data show the importance and influence of the TME of PDAC in anti-cancer drug screening and angiogenic studies, for which we offer high-throughput in vitro 3D models that consider intra- and intertumoral heterogeneity.

## 5. Conclusions

We have established a panel of triple co-culture spheroid models that capture the complexity and heterogeneity of the pancreatic ductal adenocarcinoma tumor microenvironment. Unlike other alternatives, we were able to achieve this with a simple, inexpensive, and easily reproducible method while ensuring that each cell population was present in a clinically relevant number. We showed the value of our triple co-culture spheroids for high-throughput drug screening and angiogenesis evaluation; however, our model is not limited to these applications. We have provided a valuable tool for understanding this devastating disease and for exploring new treatment strategies with higher clinical translatability.

## Figures and Tables

**Table 1:** Technical information on the image acquisition of triple co-culture spheroids using the Spark^®^ Cyto (Tecan).

Cell Line	Excitation	Emission	LED Intensity (%)	Exposure Time (ms)
BxPC-3	543–566	580–611	25	300
MiaPaCa-2	543–566	580–611	20	60
RLT-PSC	461–487	500–530	10	50
hPSC21	461–487	500–530	20	80
HMEC-1	381–400	414–450	15	45

**Table 2:** Overview of the different proposed spheroid models, their seeding ratios, most visible characteristics, drug responses, and angiogenic responses.

Spheroid Model	Seeding Ratio	Seeding Density (Cells)	Feature	Drug Response	Angiogenic Activity
BxPC-3:RLT-PSC:HMEC-1	7:2:4	5000	Dense	Low	High
BxPC-3:hPSC21:HMEC-1	6:5:3	7000	High fibrotic shield	High	Low
MiaPaCa-2:RLT-PSC:HMEC-1	6:3:3	3500	Invasive	High	Low
MiaPaCa-2:hPSC21:HMEC-1	5:6:4	4500	Fibrotic shield	High	High

## Data Availability

The datasets used and/or analyzed during the current study are available from the corresponding author upon reasonable request.

## Supplementary Materials

The following supporting information can be downloaded at https://www.mdpi.com/article/10.3390/cells14060450/s1: Figure S1: STR profile of BxPC-3; Figure S2: STR profile of HMEC-1; Figure S3: STR profile of hPSC21; Figure S4: STR profile of MiaPaCa-2; Figure S5: STR profile of RLT-PSC; Figure S6: Initial optimization process of triple co-culture spheroids; Figure S7: MCDB131 is the most suitable medium for spheroid culture; Figure S8: DMEM with VEGF/FGF supplements is not able to provide sufficient growth factors for HMEC-1 growth; Figure S9: Flow cytometry gating strategy; Figure S10: Quantitative flow cytometric data of triple co-culture spheroid ratios that were not selected as optimal; Figure S11: H&E and immunohistochemical staining of representative PDAC samples, illustrating typical characteristics of the PDAC.

## Abbreviations

The following abbreviations are used in this manuscript:

PDAC	Pancreatic ductal adenocarcinoma
TME	Tumor microenvironment
PSC	Pancreatic stellate cell
EC	Endothelial cell
TCC	Triple co-culture
CAF	Cancer-associated fibroblast
2D	Two-dimensional
3D	Three-dimensional
ECM	Extracellular matrix
PDO	Patient-derived organoid
AUC	Area under the curve
STR	Short tandem repeat

## References

[1] Rahib L., Smith B.D., Aizenberg R., Rosenzweig A.B., Fleshman J.M., Matrisian L.M. (2014). Projecting cancer incidence and deaths to 2030: The unexpected burden of thyroid, liver, and pancreas cancers in the United States. Cancer Res..

[2] Hasan S., Jacob R., Manne U., Paluri R. (2019). Advances in pancreatic cancer biomarkers. Oncol. Rev..

[3] de Sousa Cavalcante L., Monteiro G. (2014). Gemcitabine: Metabolism and molecular mechanisms of action, sensitivity and chemoresistance in pancreatic cancer. Eur. J. Pharmacol..

[4] Zeisberg E.M., Potenta S., Xie L., Zeisberg M., Kalluri R. (2007). Discovery of endothelial to mesenchymal transition as a source for carcinoma-associated fibroblasts. Cancer Res..

[5] Platel V., Faure S., Corre I., Clere N. (2019). Endothelial-to-Mesenchymal Transition (EndoMT): Roles in Tumorigenesis, Metastatic Extravasation and Therapy Resistance. J. Oncol..

[6] Verloy R., Privat-Maldonado A., Smits E., Bogaerts A. (2020). Cold Atmospheric Plasma Treatment for Pancreatic Cancer—The Importance of Pancreatic Stellate Cells. Cancers.

[7] Hayashi H., Higashi T., Miyata T., Yamashita Y.I., Baba H. (2021). Recent advances in precision medicine for pancreatic ductal adenocarcinoma. Ann. Gastroenterol. Surg..

[8] Nusrat F., Khanna A., Jain A., Jiang W., Lavu H., Yeo C.J., Bowne W., Nevler A. (2024). The Clinical Implications of *KRAS* Mutations and Variant Allele Frequencies in Pancreatic Ductal Adenocarcinoma. J. Clin. Med..

[9] Stefanoudakis D., Frountzas M., Schizas D., Michalopoulos N.V., Drakaki A., Toutouzas K.G. (2024). Significance of *TP53*, *CDKN2A*, *SMAD4* and *KRAS* in Pancreatic Cancer. Curr. Issues Mol. Biol..

[10] Racu M.L., Bernardi D., Chaouche A., Zindy E., Navez J., Loi P., Maris C., Closset J., Van Laethem J.L., Decaestecker C. (2023). *SMAD4* Positive Pancreatic Ductal Adenocarcinomas Are Associated with Better Outcomes in Patients Receiving FOLFIRINOX-Based Neoadjuvant Therapy. Cancers.

[11] McCubrey J.A., Yang L.V., Abrams S.L., Steelman L.S., Follo M.Y., Cocco L., Ratti S., Martelli A.M., Augello G., Cervello M. (2022). Effects of *TP53* Mutations and miRs on Immune Responses in the Tumor Microenvironment Important in Pancreatic Cancer Progression. Cells.

[12] Peng J., Sun B.-F., Chen C.-Y., Zhou J.-Y., Chen Y.-S., Chen H., Liu L., Huang D., Jiang J., Cui G.-S. (2019). Single-cell RNA-seq highlights intra-tumoral heterogeneity and malignant progression in pancreatic ductal adenocarcinoma. Cell Res..

[13] Wang Y., Liang Y., Xu H., Zhang X., Mao T., Cui J., Yao J., Wang Y., Jiao F., Xiao X. (2021). Single-cell analysis of pancreatic ductal adenocarcinoma identifies a novel fibroblast subtype associated with poor prognosis but better immunotherapy response. Cell Discov..

[14] Werba G., Weissinger D., Kawaler E.A., Zhao E., Kalfakakou D., Dhara S., Wang L., Lim H.B., Oh G., Jing X. (2023). Single-cell RNA sequencing reveals the effects of chemotherapy on human pancreatic adenocarcinoma and its tumor microenvironment. Nat. Commun..

[15] Ye B., Wang Q., Zhu X., Zeng L., Luo H., Xiong Y., Li Q., Zhu Q., Zhao S., Chen T. (2023). Single-cell RNA sequencing identifies a novel proliferation cell type affecting clinical outcome of pancreatic ductal adenocarcinoma. Front. Oncol..

[16] Yu Y., Yang G., Huang H., Fu Z., Cao Z., Zheng L., You L., Zhang T. (2021). Preclinical models of pancreatic ductal adenocarcinoma: Challenges and opportunities in the era of precision medicine. J. Exp. Clin. Cancer Res..

[17] Cros J., Raffenne J., Couvelard A., Pote N. (2018). Tumor Heterogeneity in Pancreatic Adenocarcinoma. Pathobiology.

[18] Wang Y., Jeon H. (2022). 3D cell cultures toward quantitative high-throughput drug screening. Trends Pharmacol. Sci..

[19] Parasrampuria D.A., Benet L.Z., Sharma A. (2018). Why Drugs Fail in Late Stages of Development: Case Study Analyses from the Last Decade and Recommendations. AAPS J..

[20] Brüningk S.C., Rivens I., Box C., Oelfke U., ter Haar G. (2020). 3D tumour spheroids for the prediction of the effects of radiation and hyperthermia treatments. Sci. Rep..

[21] Pape J., Emberton M., Cheema U. (2021). 3D Cancer Models: The Need for a Complex Stroma, Compartmentalization and Stiffness. Front. Bioeng. Biotechnol..

[22] Tomas-Bort E., Kieler M., Sharma S., Candido J.B., Loessner D. (2020). 3D approaches to model the tumor microenvironment of pancreatic cancer. Theranostics.

[23] Ware M.J., Keshishian V., Law J.J., Ho J.C., Favela C.A., Rees P., Smith B., Mohammad S., Hwang R.F., Rajapakshe K. (2016). Generation of an in vitro 3D PDAC stroma rich spheroid model. Biomaterials.

[24] Drifka C.R., Loeffler A.G., Esquibel C.R., Weber S.M., Eliceiri K.W., Kao W.J. (2016). Human pancreatic stellate cells modulate 3D collagen alignment to promote the migration of pancreatic ductal adenocarcinoma cells. Biomed. Microdevices.

[25] Lazzari G., Nicolas V., Matsusaki M., Akashi M., Couvreur P., Mura S. (2018). Multicellular spheroid based on a triple co-culture: A novel 3D model to mimic pancreatic tumor complexity. Acta Biomater..

[26] Steinberg E., Orehov N., Tischenko K., Schwob O., Zamir G., Hubert A., Manevitch Z., Benny O. (2020). Rapid Clearing for High Resolution 3D Imaging of Ex Vivo Pancreatic Cancer Spheroids. Int. J. Mol. Sci..

[27] Yang H., Sun L., Liu M., Mao Y. (2018). Patient-derived organoids: A promising model for personalized cancer treatment. Gastroenterol. Rep..

[28] Ohlund D., Handly-Santana A., Biffi G., Elyada E., Almeida A.S., Ponz-Sarvise M., Corbo V., Oni T.E., Hearn S.A., Lee E.J. (2017). Distinct populations of inflammatory fibroblasts and myofibroblasts in pancreatic cancer. J. Exp. Med..

[29] Tsai S., McOlash L., Palen K., Johnson B., Duris C., Yang Q., Dwinell M.B., Hunt B., Evans D.B., Gershan J. (2018). Development of primary human pancreatic cancer organoids, matched stromal and immune cells and 3D tumor microenvironment models. BMC Cancer.

[30] Rae C., Amato F., Braconi C. (2021). Patient-Derived Organoids as a Model for Cancer Drug Discovery. Int. J. Mol. Sci..

[31] Foo M.A., You M., Chan S.L., Sethi G., Bonney G.K., Yong W.P., Chow E.K., Fong E.L.S., Wang L., Goh B.C. (2022). Clinical translation of patient-derived tumour organoids- bottlenecks and strategies. Biomark. Res..

[32] Jesnowski R., Furst D., Ringel J., Chen Y., Schrodel A., Kleeff J., Kolb A., Schareck W.D., Lohr M. (2005). Immortalization of pancreatic stellate cells as an in vitro model of pancreatic fibrosis: Deactivation is induced by matrigel and N-acetylcysteine. Lab. Investig..

[33] Hamada S., Masamune A., Takikawa T., Suzuki N., Kikuta K., Hirota M., Hamada H., Kobune M., Satoh K., Shimosegawa T. (2012). Pancreatic stellate cells enhance stem cell-like phenotypes in pancreatic cancer cells. Biochem. Biophys. Res. Commun..

[34] Arndt S., Unger P., Berneburg M., Bosserhoff A.K., Karrer S. (2018). Cold atmospheric plasma (CAP) activates angiogenesis-related molecules in skin keratinocytes, fibroblasts and endothelial cells and improves wound angiogenesis in an autocrine and paracrine mode. J. Dermatol. Sci..

[35] Xu Z., Vonlaufen A., Phillips P.A., Fiala-Beer E., Zhang X., Yang L., Biankin A.V., Goldstein D., Pirola R.C., Wilson J.S. (2010). Role of pancreatic stellate cells in pancreatic cancer metastasis. Am. J. Pathol..

[36] Van den Eynde A., Gehrcken L., Verhezen T., Lau H.W., Hermans C., Lambrechts H., Flieswasser T., Quatannens D., Roex G., Zwaenepoel K. (2024). IL-15-secreting CAR natural killer cells directed toward the pan-cancer target CD70 eliminate both cancer cells and cancer-associated fibroblasts. J. Hematol. Oncol..

[37] Lee H., Kang K.T. (2018). Advanced tube formation assay using human endothelial colony forming cells for in vitro evaluation of angiogenesis. Korean J. Physiol. Pharmacol..

[38] Han S.J., Kwon S., Kim K.S. (2021). Challenges of applying multicellular tumor spheroids in preclinical phase. Cancer Cell Int..

[39] Belvedere R., Bizzarro V., Popolo A., Dal Piaz F., Vasaturo M., Picardi P., Parente L., Petrella A. (2014). Role of intracellular and extracellular annexin A1 in migration and invasion of human pancreatic carcinoma cells. BMC Cancer.

[40] Svirshchevskaya E., Doronina E., Grechikhina M., Matushevskaya E., Kotsareva O., Fattakhova G., Sapozhnikov A., Felix K. (2019). Characteristics of multicellular tumor spheroids formed by pancreatic cells expressing different adhesion molecules. Life Sci..

[41] Kapalczynska M., Kolenda T., Przybyla W., Zajaczkowska M., Teresiak A., Filas V., Ibbs M., Blizniak R., Luczewski L., Lamperska K. (2018). 2D and 3D cell cultures—A comparison of different types of cancer cell cultures. Arch. Med. Sci..

[42] Longati P., Jia X., Eimer J., Wagman A., Witt M.R., Rehnmark S., Verbeke C., Toftgard R., Lohr M., Heuchel R.L. (2013). 3D pancreatic carcinoma spheroids induce a matrix-rich, chemoresistant phenotype offering a better model for drug testing. BMC Cancer.

[43] Pan X., Arumugam T., Yamamoto T., Levin P.A., Ramachandran V., Ji B., Lopez-Berestein G., Vivas-Mejia P.E., Sood A.K., McConkey D.J. (2008). Nuclear factor-kappaB p65/relA silencing induces apoptosis and increases gemcitabine effectiveness in a subset of pancreatic cancer cells. Clin. Cancer Res..

[44] Maietta I., Martinez-Perez A., Alvarez R., De Lera A.R., Gonzalez-Fernandez A., Simon-Vazquez R. (2022). Synergistic Antitumoral Effect of Epigenetic Inhibitors and Gemcitabine in Pancreatic Cancer Cells. Pharmaceuticals.

[45] Patki M., Saraswat A., Bhutkar S., Dukhande V., Patel K. (2021). In vitro assessment of a synergistic combination of gemcitabine and zebularine in pancreatic cancer cells. Exp. Cell Res..

[46] Nilendu P., Sarode S.C., Jahagirdar D., Tandon I., Patil S., Sarode G.S., Pal J.K., Sharma N.K. (2018). Mutual concessions and compromises between stromal cells and cancer cells: Driving tumor development and drug resistance. Cell. Oncol..

[47] Le Compte M., De La Hoz E.C., Peeters S., Fortes F.R., Hermans C., Domen A., Smits E., Lardon F., Vandamme T., Lin A. (2023). Single-organoid analysis reveals clinically relevant treatment-resistant and invasive subclones in pancreatic cancer. NPJ Precis. Oncol..

[48] Nishida N., Yano H., Nishida T., Kamura T., Kojiro M. (2006). Angiogenesis in cancer. Vasc. Health Risk Manag..

[49] Mercanti L., Sindaco M., Mazzone M., Di Marcantonio M.C., Piscione M., Muraro R., Mincione G. (2023). PDAC, the Influencer Cancer: Cross-Talk with Tumor Microenvironment and Connected Potential Therapy Strategies. Cancers.

[50] Barrera L.N., Evans A., Lane B., Brumskill S., Oldfield F.E., Campbell F., Andrews T., Lu Z., Perez-Mancera P.A., Liloglou T. (2020). Fibroblasts from Distinct Pancreatic Pathologies Exhibit Disease-Specific Properties. Cancer Res..

[51] Zhu Y., Kang E., Wilson M., Basso T., Chen E., Yu Y., Li Y.-R. (2022). 3D Tumor Spheroid and Organoid to Model Tumor Microenvironment for Cancer Immunotherapy. Organoids.

