# Supplementary material for: Capturing the Heterogeneity of the PDAC Tumor Microenvironment: Novel Triple Co-Culture Spheroids for Drug Screening and Angiogenic Evaluation
Source: Cells. 2025 Mar 18;14(6):450. doi: 10.3390/cells14060450 (PMC11940881; doi:10.3390/cells14060450)
Supplement: Supplementary file 1 [file cells-14-00450-s001.zip › cells-3504694-supplementary.pdf]

cells

Capturing the heterogeneity of the PDAC tumor microenvironment: novel triple co-culture spheroids for drug screening and angiogenic evaluation

Ruben Verloy[1,2,*], Angela Privat-Maldonado[1,2], Jonas Van Audenaerde[2], Sophie Rovers[2], Hannah Zaryouh[2], Jorrit De Waele[2], Delphine Quatannens[2], Dieter Peeters[2,3], Geert Roeyen[2,4], Christophe Deben[2], Evelien Smits[2,†] and Annemie Bogaerts[1,†]

1 Research group PLASMANT, Department of Chemistry, University of Antwerp, Belgium
2 Center for Oncological Research (CORE), Integrated Personalized and Precision Oncology Network (IPPON), University of Antwerp, Belgium
3 Department of Pathology, University Hospital Antwerp/University of Antwerp, Antwerp, Belgium
4 Department of Hepatobiliary Transplantation and Endocrine Surgery, University Hospital Antwerp (UZA), Antwerp, Belgium
* Corresponding author: ruben.verloy@uantwerpen.be
† These authors contributed equally to this work.

Dye: Blue - 9 peaks - DG_Ruben_BxPC_3-O09-015.fsa

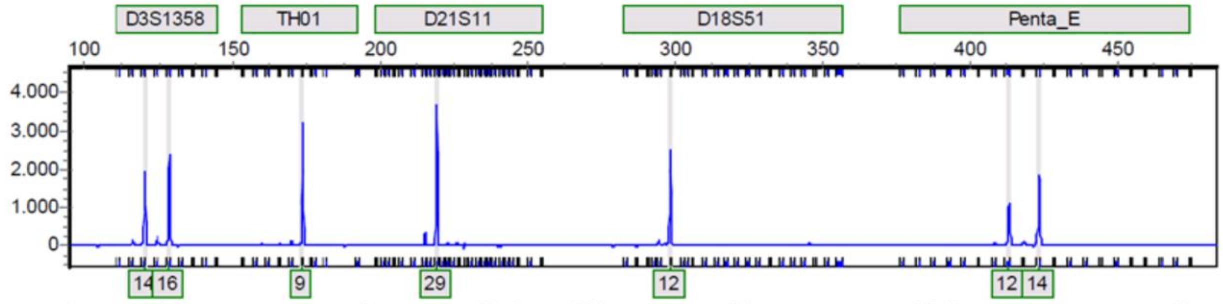

No	Size	Height	Area	Marker	Allele	Difference	Quality	Score	Allele Comments	Sample Comments
1	120.5	1941	9895	D3S1358	14	0.18	Pass	327.8	[<Confirmed><Inserted>]	
2	128.6	2031	11690	D3S1358	16	0.06	Pass	288.6	[<Confirmed><Inserted>]	
3	174.0	3232	14603	TH01	9	0.00	Pass	500.0	[<Confirmed><Inserted>]	
4	215.2	349	1714	D21S11	28	0.14	Pass	27.3	[<Deleted>]	
5	219.2	3665	17547	D21S11	29	0.38	Pass	500.0		
6	298.3	2502	13525	D18S51	OL	1.00	Undetermined	480.8	[<Deleted>]	
7	298.8	386	14853	D18S51	12	0.15	Pass	0.0	[<Confirmed><Inserted>]	
8	413.1	1105	7538	Penta_E	12	0.08	Pass	98.5		
9	423.4	1839	12642	Penta_E	14	0.04	Pass	210.8		

Dye: Green - 8 peaks - DG_Ruben_BxPC_3-O09-015.fsa

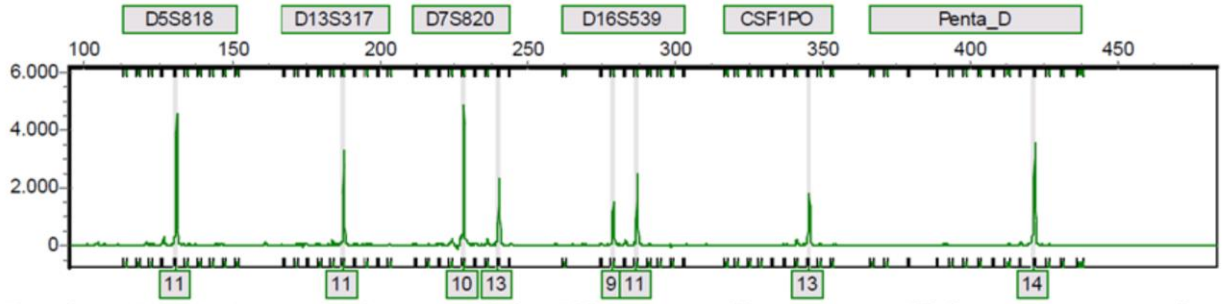

No	Size	Height	Area	Marker	Allele	Difference	Quality	Score	Allele Comments	Sample Comments
1	131.1	4559	22487	D5S818	11	0.29	Pass	500.0	[<Confirmed><Inserted>]	
2	187.9	3278	15302	D13S317	11	0.07	Pass	500.0	[<Confirmed><Inserted>]	
3	228.5	4824	23964	D7S820	10	0.00	Pass	500.0		
4	240.4	2320	11844	D7S820	13	0.07	Pass	320.1		
5	279.1	1465	7783	D16S539	9	0.06	Pass	169.2		
6	287.2	2455	12832	D16S539	11	0.30	Pass	398.6		
7	345.3	1827	10761	CSF1PO	13	0.18	Pass	220.9		
8	421.8	3528	24405	Penta_D	14	0.02	Pass	427.5		

Dye: Yellow - 8 peaks - DG_Ruben_BxPC_3-O09-015.fsa

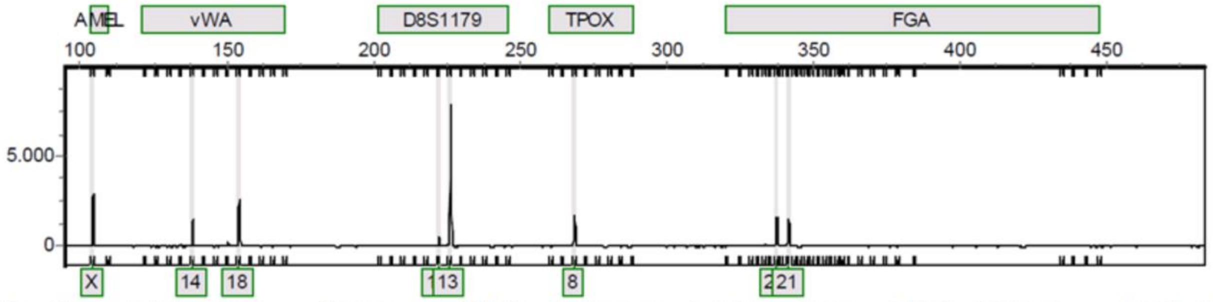

No	Size	Height	Area	Marker	Allele	Difference	Quality	Score	Allele Comments	Sample Comments
1	104.3	2903	14214	AMEL	X	0.14	Pass	500.0	[<Confirmed><Inserted>]	
2	138.4	1584	7666	vWA	14	0.05	Pass	330.2	[<Confirmed><Inserted>]	
3	154.3	2673	12985	vWA	18	0.00	Pass	500.0	[<Confirmed><Inserted>]	
4	222.4	524	2437	D8S1179	12	0.21	Pass	75.8		
5	226.4	7832	38724	D8S1179	13	0.29	Pass	500.0		
6	268.6	1696	8712	TPOX	8	0.04	Pass	375.8		
7	337.6	1634	9159	FGA	20	0.23	Pass	314.2		
8	341.7	1512	8562	FGA	21	0.18	Pass	244.6		

Supplementary Figure S1. Short tandem repeats (STR) profile of BxPC-3.

Dye: Blue - 9 peaks - DG_Ruben_HMEC_1-F09-005.fsa

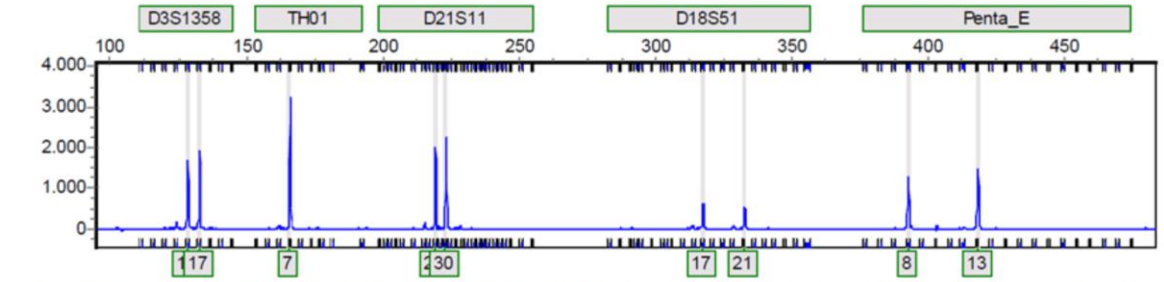

No	Size	Height	Area	Marker	Allele	Difference	Quality	Score	Allele Comments	Sample Comments
1	128.5	1682	8665	D3S1358	16	0.04	Pass	327.6	[<Confirmed><Inserted>]	
2	133.0	1591	9834	D3S1358	17	0.25	Pass	225.9	[<Confirmed><Inserted>]	
3	166.0	3212	14702	TH01	7	0.00	Pass	500.0	[<Confirmed><Inserted>]	
4	219.3	2010	9312	D21S11	29	0.28	Pass	481.0		
5	223.3	2249	10707	D21S11	30	0.23	Pass	500.0		
6	317.4	622	3381	D18S51	17	0.09	Pass	68.6		
7	332.6	543	2931	D18S51	21	0.19	Pass	55.6		
8	392.7	1269	7619	Penta_E	8	0.09	Pass	171.1		
9	418.4	1464	9353	Penta_E	13	0.15	Pass	190.7		

Dye: Green - 11 peaks - DG_Ruben_HMEC_1-F09-005.fsa

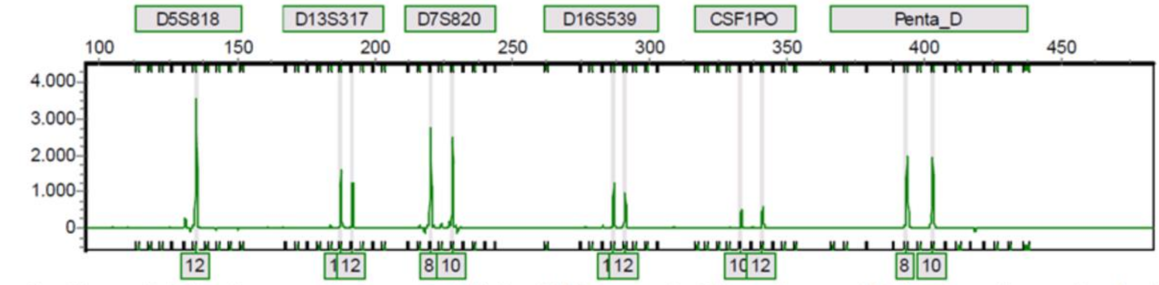

No	Size	Height	Area	Marker	Allele	Difference	Quality	Score	Allele Comments	Sample Comments
1	135.1	3515	17396	D5S818	12	0.20	Pass	500.0	[<Confirmed><Inserted>]	
2	188.0	1300	7765	D13S317	11	0.17	Pass	104.4	[<Confirmed><Inserted>]	
3	192.0	1265	5991	D13S317	12	0.33	Pass	158.8	[<Confirmed><Inserted>]	
4	220.4	2759	13563	D7S820	8	0.01	Pass	466.3		
5	228.4	2506	12473	D7S820	10	0.10	Pass	399.8		
6	287.1	1248	6382	D16S539	11	0.20	Pass	140.8		
7	291.1	993	5081	D16S539	12	0.16	Pass	98.4		
8	333.3	490	2539	CSF1PO	10	0.36	Pass	35.3		
9	341.4	610	3482	CSF1PO	12	0.18	Pass	42.9		
10	393.7	1995	12340	Penta_D	8	0.13	Pass	231.5		
11	403.2	1915	11838	Penta_D	10	0.05	Pass	217.0		

Dye: Yellow - 10 peaks - DG_Ruben_HMEC_1-F09-005.fsa

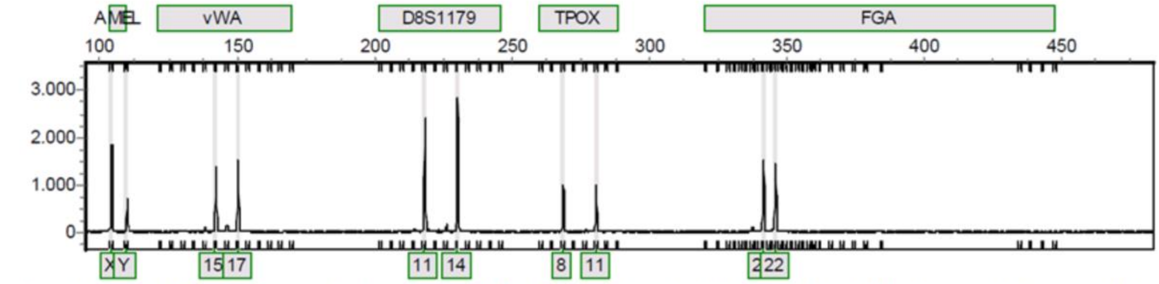

No	Size	Height	Area	Marker	Allele	Difference	Quality	Score	Allele Comments	Sample Comments
1	104.3	1850	9274	AMEL	X	0.14	Pass	499.3	[<Confirmed><Inserted>]	
2	110.0	706	3394	AMEL	Y	0.07	Pass	133.2	[<Confirmed><Inserted>]	
3	142.3	1360	6555	vWA	15	0.02	Pass	343.9	[<Confirmed><Inserted>]	
4	150.3	1524	7245	vWA	17	0.02	Pass	408.5	[<Confirmed><Inserted>]	
5	218.3	2412	11120	D8S1179	11	0.06	Pass	500.0		
6	230.3	2821	13308	D8S1179	14	0.16	Pass	500.0		
7	268.7	975	4918	TPOX	8	0.06	Pass	198.8		
8	280.7	1000	4992	TPOX	11	0.03	Pass	209.3		
9	341.7	1528	8414	FGA	21	0.18	Pass	334.8		
10	345.8	1440	7960	FGA	22	0.14	Pass	306.0		

Supplementary Figure S2. STR profile of HMEC-1.

Dye: Blue - 9 peaks - DG_Ruben_hPSC21-D09-003.fsa

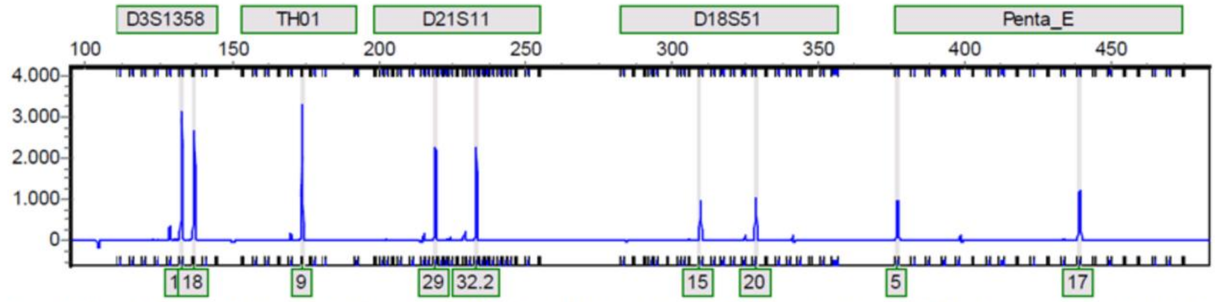

No	Size	Height	Area	Marker	Allele	Difference	Quality	Score	Allele Comments	Sample Comments
1	132.9	2977	15864	D3S1358	17	0.15	Pass	500.0	[<Confirmed><Inserted>]	
2	137.0	2641	12882	D3S1358	18	0.14	Pass	500.0	[<Confirmed><Inserted>]	
3	174.1	2782	15092	TH01	9	0.10	Pass	500.0	[<Confirmed><Inserted>]	
4	219.3	2250	10632	D21S11	29	0.28	Pass	493.7		
5	233.3	2276	10928	D21S11	32.2	0.18	Pass	492.1		
6	309.8	989	5244	D18S51	15	0.49	Pass	145.8		
7	328.8	1075	5734	D18S51	20	0.20	Pass	162.8		
8	377.1	984	5847	Penta_E	5	0.10	Pass	118.8		
9	439.3	1210	8172	Penta_E	17	0.07	Pass	148.2		

Dye: Green - 10 peaks - DG_Ruben_hPSC21-D09-003.fsa

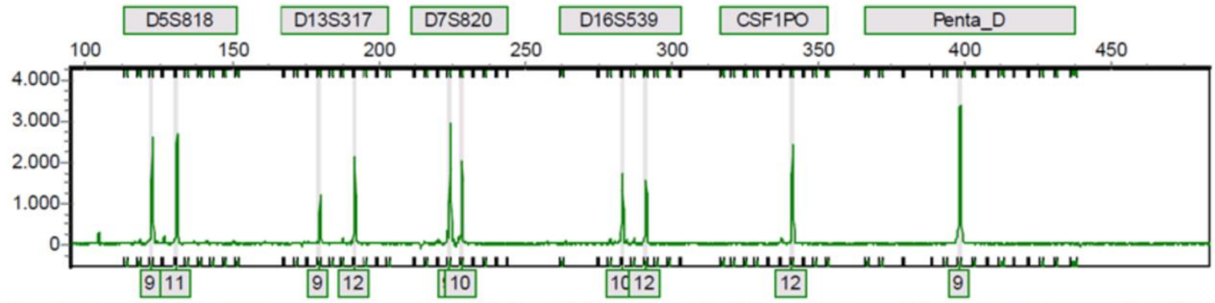

No	Size	Height	Area	Marker	Allele	Difference	Quality	Score	Allele Comments	Sample Comments
1	122.8	2362	12865	D5S818	9	0.34	Pass	326.2	[<Confirmed><Inserted>]	
2	131.2	2411	13235	D5S818	11	0.39	Pass	327.7	[<Confirmed><Inserted>]	
3	180.0	1213	5522	D13S317	9	0.24	Pass	124.0	[<Confirmed><Inserted>]	
4	191.8	2132	9674	D13S317	12	0.13	Pass	305.3	[<Confirmed><Inserted>]	
5	224.5	2933	14300	D7S820	9	0.09	Pass	438.2		
6	228.4	2009	10085	D7S820	10	0.10	Pass	248.1		
7	283.1	1731	8919	D16S539	10	0.08	Pass	207.5		
8	291.2	1566	8205	D16S539	12	0.06	Pass	174.9		
9	341.4	2403	13234	CSF1PO	12	0.18	Pass	328.4		
10	398.5	3351	20917	Penta_D	9	0.01	Pass	500.0		

Dye: Yellow - 7 peaks - DG_Ruben_hPSC21-D09-003.fsa

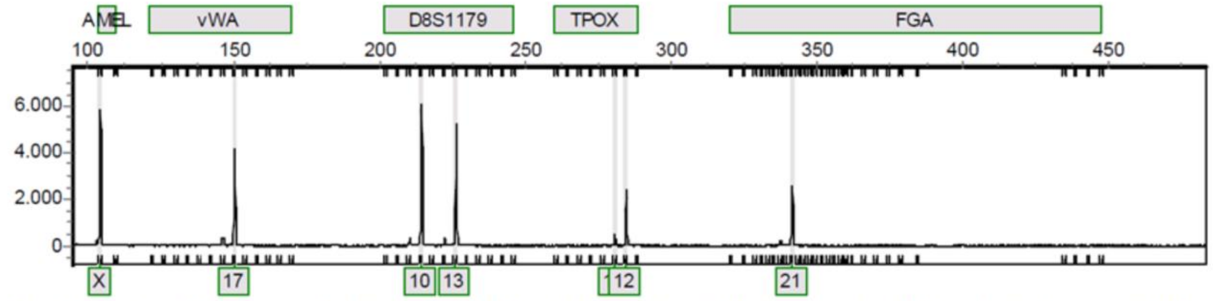

No	Size	Height	Area	Marker	Allele	Difference	Quality	Score	Allele Comments	Sample Comments
1	104.2	5837	28216	AMEL	X	0.04	Pass	500.0	[<Confirmed><Inserted>]	
2	150.3	4132	19573	vWA	17	0.02	Pass	500.0	[<Confirmed><Inserted>]	
3	214.4	6008	27895	D8S1179	10	0.12	Pass	500.0		
4	226.3	5238	24110	D8S1179	13	0.19	Pass	500.0		
5	280.7	488	2510	TPOX	11	0.03	Pass	67.2		
6	284.7	2361	11743	TPOX	12	0.02	Pass	500.0		
7	341.7	2553	13710	FGA	21	0.18	Pass	500.0		

Supplementary Figure S3. STR profile of hPSC21.

Dye: Blue - 9 peaks - DG_Ruben_MiaPACa_2-M09-013.fsa

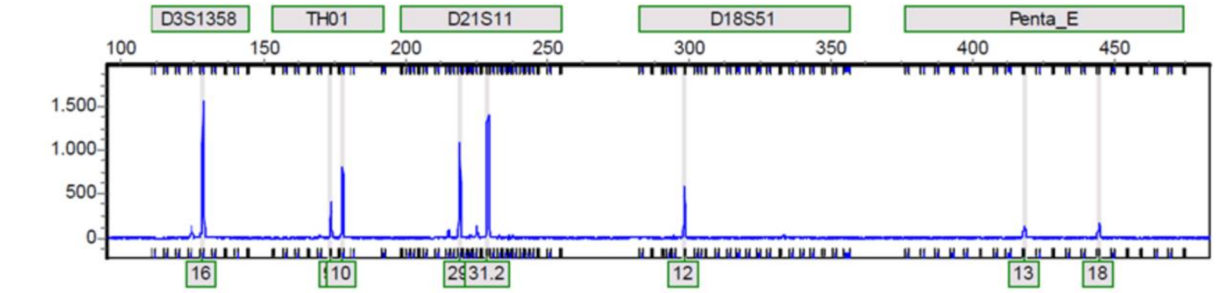

No	Size	Height	Area	Marker	Allele	Difference	Quality	Score	Allele Comments	Sample Comments
1	128.8	1551	7591	D3S1358	16	0.26	Pass	312.7	[<Confirmed><Inserted>]	
2	173.9	415	1983	TH01	9	0.10	Pass	49.6	[<Confirmed><Inserted>]	
3	177.9	807	3721	TH01	10	0.10	Pass	129.3	[<Confirmed><Inserted>]	
4	219.2	1090	5276	D21S11	29	0.38	Pass	217.2		
5	229.2	1402	6894	D21S11	31.2	0.27	Pass	304.0		
6	298.4	586	3085	D18S51	OL	1.00	Undetermined	75.6	[<Deleted>]	
7	298.7	208	3293	D18S51	12	0.25	Pass	0.0	[<Confirmed><Inserted>]	
8	418.3	142	1048	Penta_E	13	0.05	Pass	3.1	[<Confirmed>]	
9	444.6	172	1298	Penta_E	18	0.24	Pass	4.5	[<Confirmed>]	

Dye: Green - 12 peaks - DG_Ruben_MiaPACa_2-M09-013.fsa

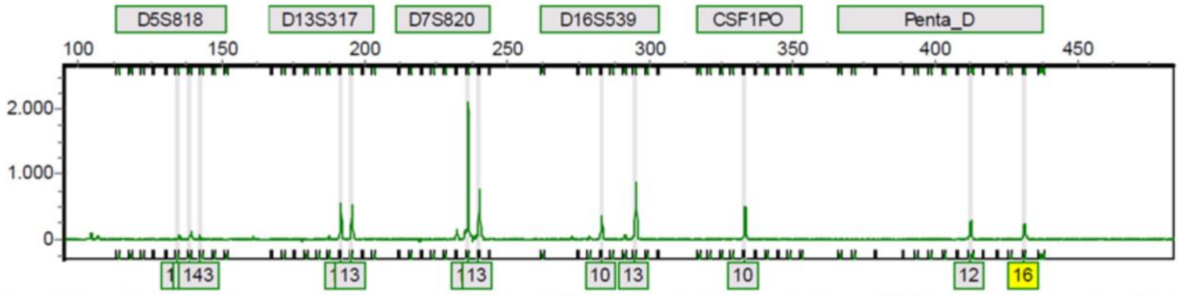

No	Size	Height	Area	Marker	Allele	Difference	Quality	Score	Allele Comments	Sample Comments
1	135.0	67	413	D5S818	12	0.10	Pass	0.9	[<Confirmed><Inserted>]	
2	139.3	140	715	D5S818	13	0.13	Pass	4.8	[<Confirmed><Inserted>]	
3	142.5	57	276	D5S818	143	0.76	Pass	1.1	[<Confirmed><Inserted>]	
4	191.8	552	2709	D13S317	12	0.13	Pass	51.9	[<Confirmed><Inserted>]	
5	195.9	525	2560	D13S317	13	0.23	Pass	48.3	[<Confirmed><Inserted>]	
6	236.4	2096	10556	D7S820	12	0.18	Pass	369.2		
7	240.4	761	3872	D7S820	13	0.07	Pass	82.9		
8	283.1	372	2030	D16S539	10	0.08	Pass	26.1		
9	295.2	866	4750	D16S539	13	0.11	Pass	100.1		
10	333.3	496	2805	CSF1PO	10	0.36	Pass	37.0		
11	412.5	275	1854	Penta_D	12	0.32	Pass	10.3		
12	431.4	244	1778	Penta_D	16	0.04	Check	6.5		

Dye: Yellow - 6 peaks - DG_Ruben_MiaPACa_2-M09-013.fsa

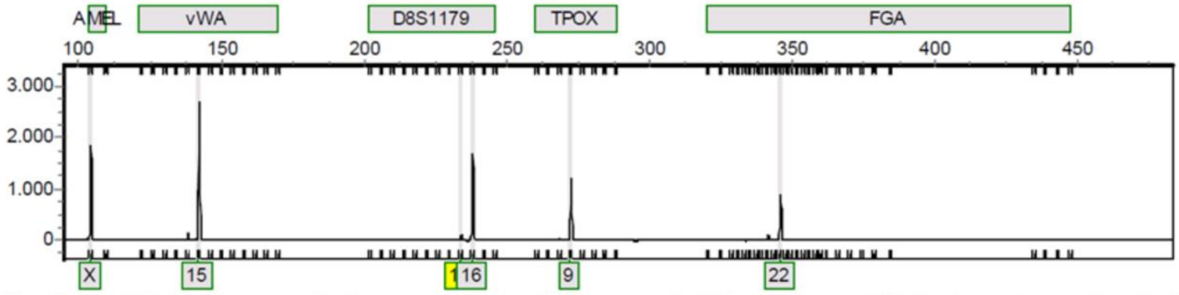

No	Size	Height	Area	Marker	Allele	Difference	Quality	Score	Allele Comments	Sample Comments
1	104.2	1852	9006	AMEL	X	0.04	Pass	404.9	[<Confirmed><Inserted>]	
2	142.3	2681	12966	vWA	15	0.02	Pass	500.0	[<Confirmed><Inserted>]	
3	234.2	123	541	D8S1179	15	0.04	Check	8.0		
4	238.2	1677	8176	D8S1179	16	0.06	Pass	397.4		
5	272.6	1195	6135	TPOX	9	0.01	Pass	228.8		
6	345.9	910	5071	FGA	22	0.24	Pass	134.8		

Supplementary Figure S4. STR profile of MiaPaCa-2.

Dye: Blue - 8 peaks - DG_Ruben_RTL_PSC-B09-001.fsa

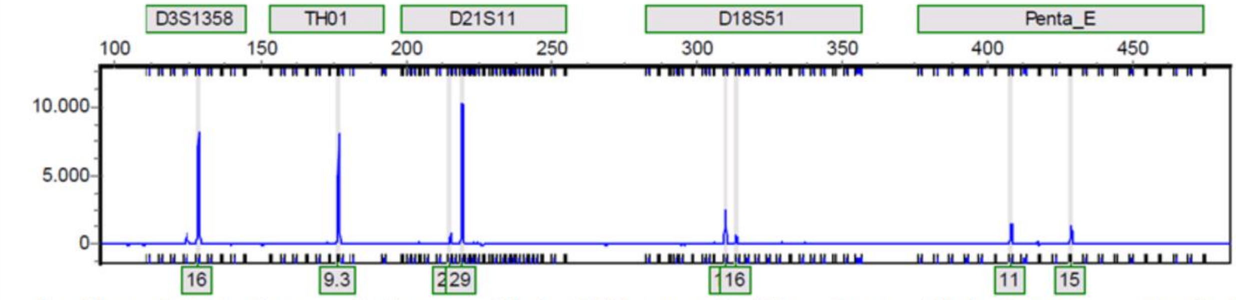

No	Size	Height	Area	Marker	Allele	Difference	Quality	Score	Allele Comments	Sample Comments
1	128.7	8207	40189	D3S1358	16	0.16	Pass	500.0	[<Confirmed><Inserted>]	
2	177.0	7001	36939	TH01	9.3	0.00	Pass	500.0	[<Confirmed><Inserted>]	
3	215.4	861	3967	D21S11	28	0.34	Pass	126.0		
4	219.3	10159	49690	D21S11	29	0.28	Pass	500.0	[<SAT (Repaired)><Confirmed>]	
5	309.9	2507	12898	D18S51	15	0.39	Pass	464.2		
6	313.8	498	3536	D18S51	16	0.47	Pass	20.2	[<Confirmed><Inserted>]	
7	408.1	1519	9089	Penta_E	11	0.02	Pass	202.0		
8	428.8	1393	9103	Penta_E	15	0.16	Pass	152.7		

Dye: Green - 8 peaks - DG_Ruben_RTL_PSC-B09-001.fsa

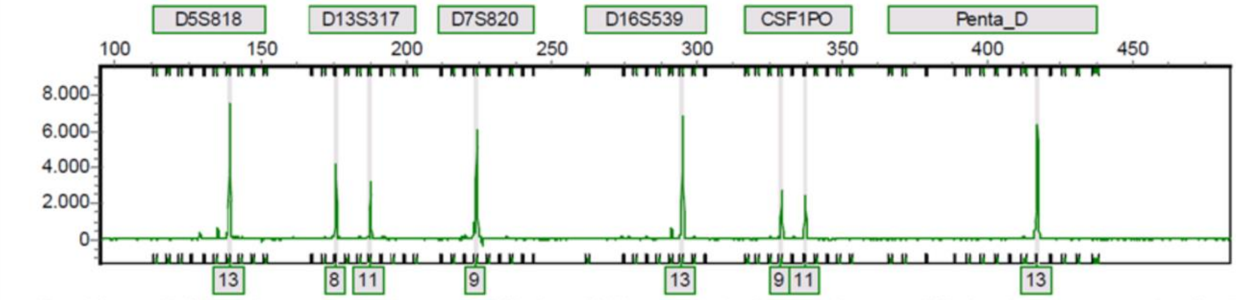

No	Size	Height	Area	Marker	Allele	Difference	Quality	Score	Allele Comments	Sample Comments
1	139.4	7479	37796	D5S818	13	0.23	Pass	500.0	[<Confirmed><Inserted>]	
2	176.0	4204	19100	D13S317	8	0.08	Pass	500.0	[<Confirmed><Inserted>]	
3	188.0	2927	14261	D13S317	11	0.17	Pass	379.2	[<Confirmed><Inserted>]	
4	224.4	6008	28658	D7S820	9	0.01	Pass	500.0		
5	295.2	6822	33962	D16S539	13	0.11	Pass	500.0		
6	329.3	2704	14098	CSF1PO	9	0.24	Pass	354.6		
7	337.3	2455	13203	CSF1PO	11	0.12	Pass	294.2		
8	417.2	6371	40378	Penta_D	13	0.07	Pass	500.0		

Dye: Yellow - 9 peaks - DG_Ruben_RTL_PSC-B09-001.fsa

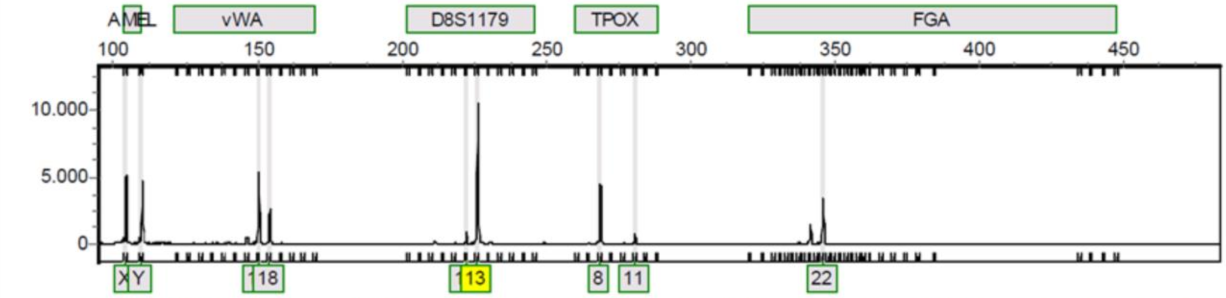

No	Size	Height	Area	Marker	Allele	Difference	Quality	Score	Allele Comments	Sample Comments
1	104.2	5029	25555	AMEL	X	0.04	Pass	500.0	[<Confirmed><Inserted>]	
2	110.0	4659	22431	AMEL	Y	0.07	Pass	500.0	[<Confirmed><Inserted>]	
3	150.3	5403	25638	vWA	17	0.02	Pass	500.0	[<Confirmed><Inserted>]	
4	154.3	2650	12277	vWA	18	0.00	Pass	500.0	[<Confirmed><Inserted>]	
5	222.3	934	4122	D8S1179	12	0.11	Pass	151.9		
6	226.3	10466	55948	D8S1179	13	0.19	Check	500.0	[<SAT (Repaired)>]	
7	268.7	4467	22335	TPOX	8	0.06	Pass	500.0		
8	280.8	746	3786	TPOX	11	0.07	Pass	101.8		
9	345.8	3376	17902	FGA	22	0.14	Pass	500.0		

Supplementary Figure S5. STR profile of RLT-PSC.

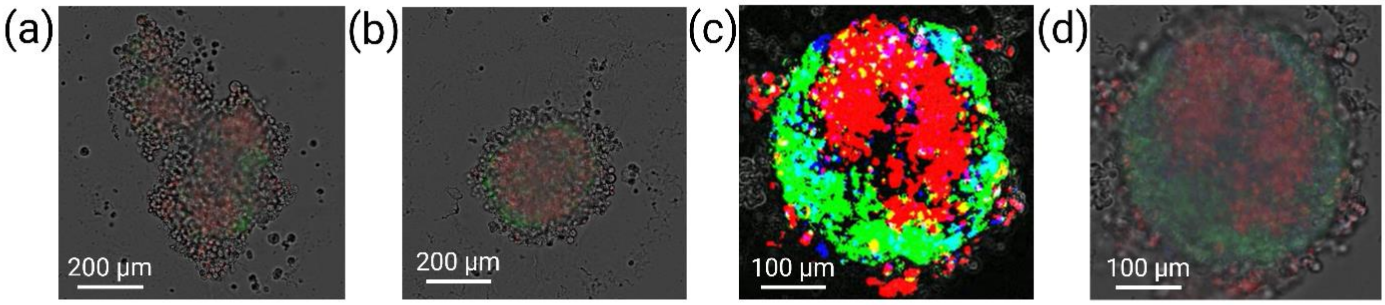

Supplementary Figure S6. Initial optimization process of triple co-culture spheroids. Representative images of (a) MiaPaCa-2:hPSC21 (1:1) spheroids which are not forming a single spheroid, and (b) MiaPaCa-2:hPSC21 (1:2) as a single spheroid. (c) Example of the masking of cancer (red), stellate (green) and endothelial (blue) cells using the Orbits image analysis software to quantify each cell type proportion on MiaPaCa-2:hPSC21:HMEC-1 (5:12:8) and (d) the original processed image of the Spark® Cyto (Tecan).

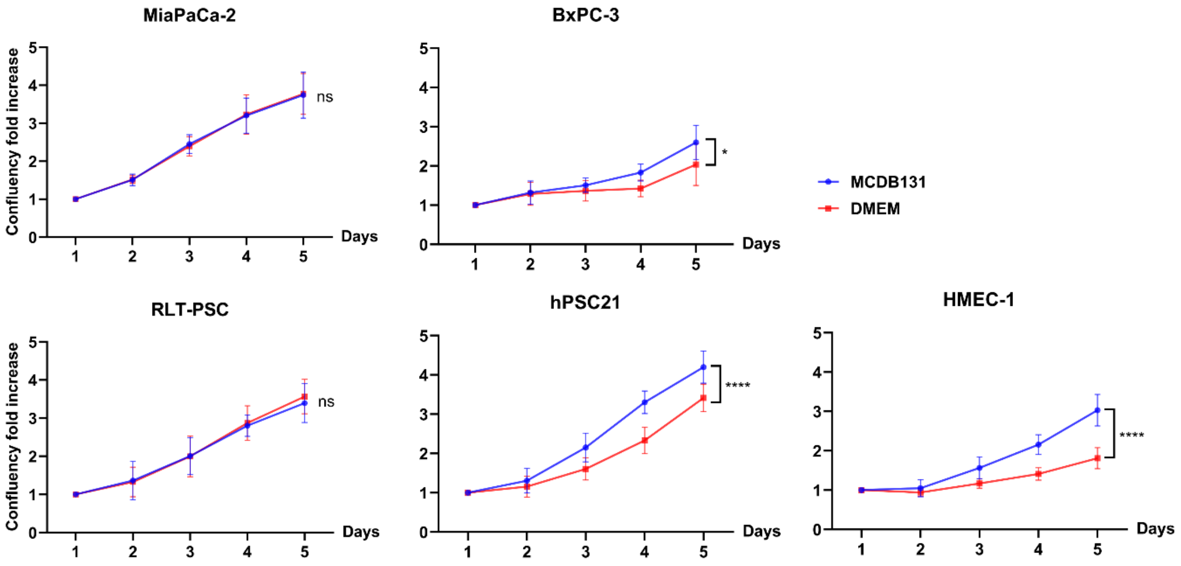

Supplementary Figure S7. MCDB131 is the most suitable medium for spheroid culture. Proliferation rate comparison of MiaPaCa-2, BxPC-3, RLT-PSC, hPSC21, and HMEC-1 in DMEM and MCDB131 is showed based on the confluence fold ratio normalized to day 1. Data are represented as mean ± SD ($n \geq 10$ from three independent experiments). Statistics were performed using two-way ANOVA with Sidak's multiple comparison test using Prism v10.1.0 (GraphPad Software, San Diego, CA, USA). * = $p \leq 0.05$; **** = $p \leq 0.0001$; ns = not significant.

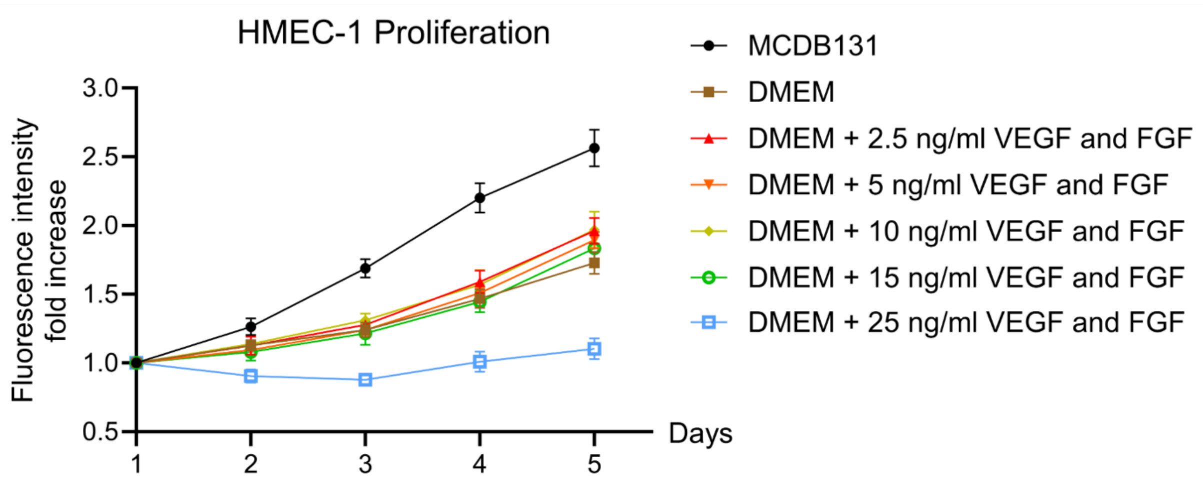

Supplementary Figure S8. DMEM with VEGF/FGF supplements is not able to provide sufficient growth factors for HMEC-1 growth. Proliferation rate comparison of HMEC-1 growth in MCDB131 vs DMEM vs DMEM with supplements, based on green fluorescence intensity fold ratio as a measure of confluency normalized to day 1. Data are represented as mean ± SD (n ≥ 9 from three independent experiments).

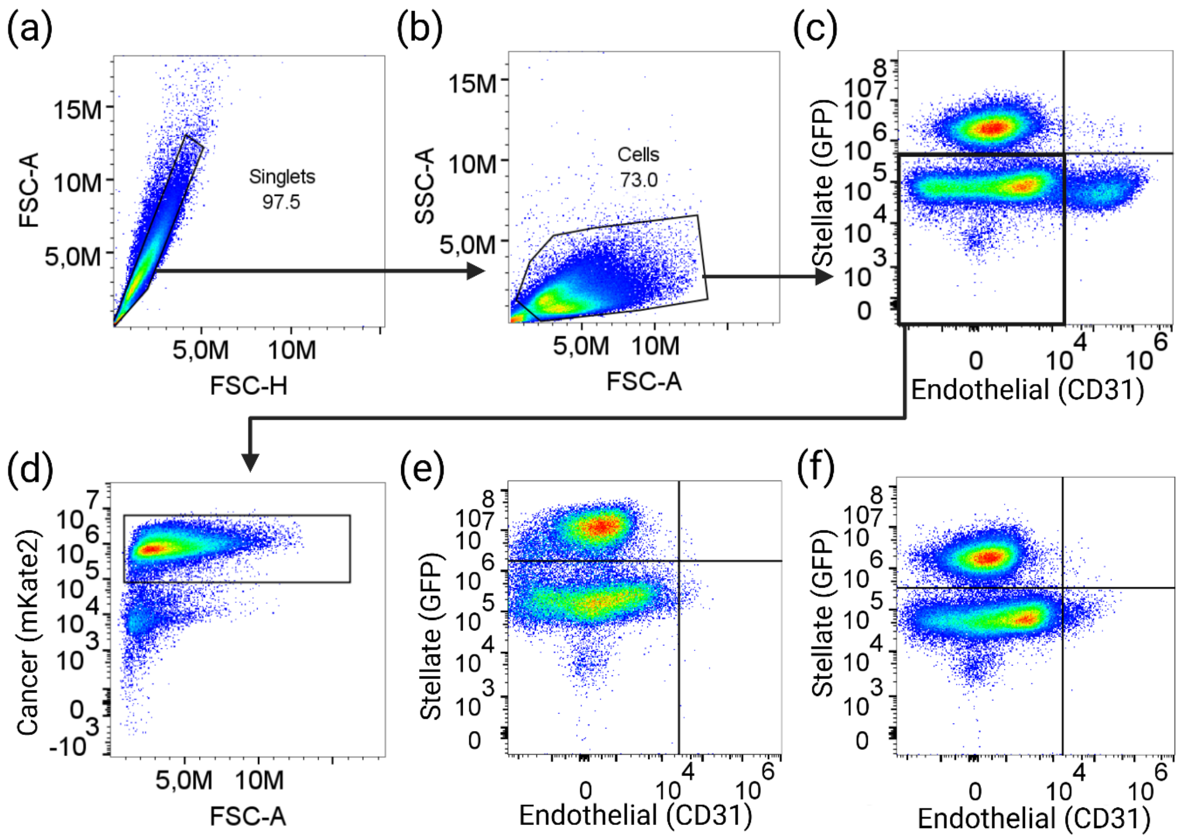

Supplementary Figure S9. Flow cytometry gating strategy. (a) Singlets, (b) cells, (c) double negative population for GFP and CD31, (d) positive population of the mKate2 (Cancer). (e) FMO control for CD31 on BxPC-3:RLT-PSC:HMEC-1 spheroids. (f) FMO control for CD31 on MiaPaCa-2:hPSC21:HMEC-1.

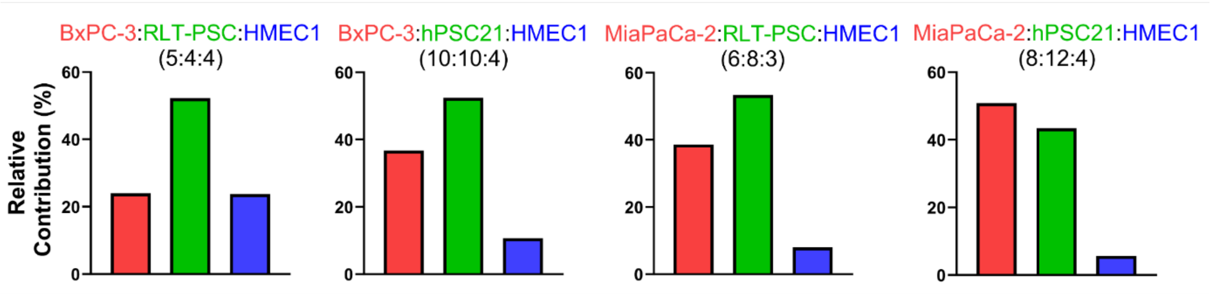

Supplementary Figure S10. Quantitative flow cytometric data of triple co-culture spheroid ratios that were not selected as optimal. Data shows the relative contribution of each cell population (each bar represents one flow cytometry measurement of a pool of 48 spheroids). Examples of TCC spheroids that were not selected due to an inaccurate representation of PDAC tumors.

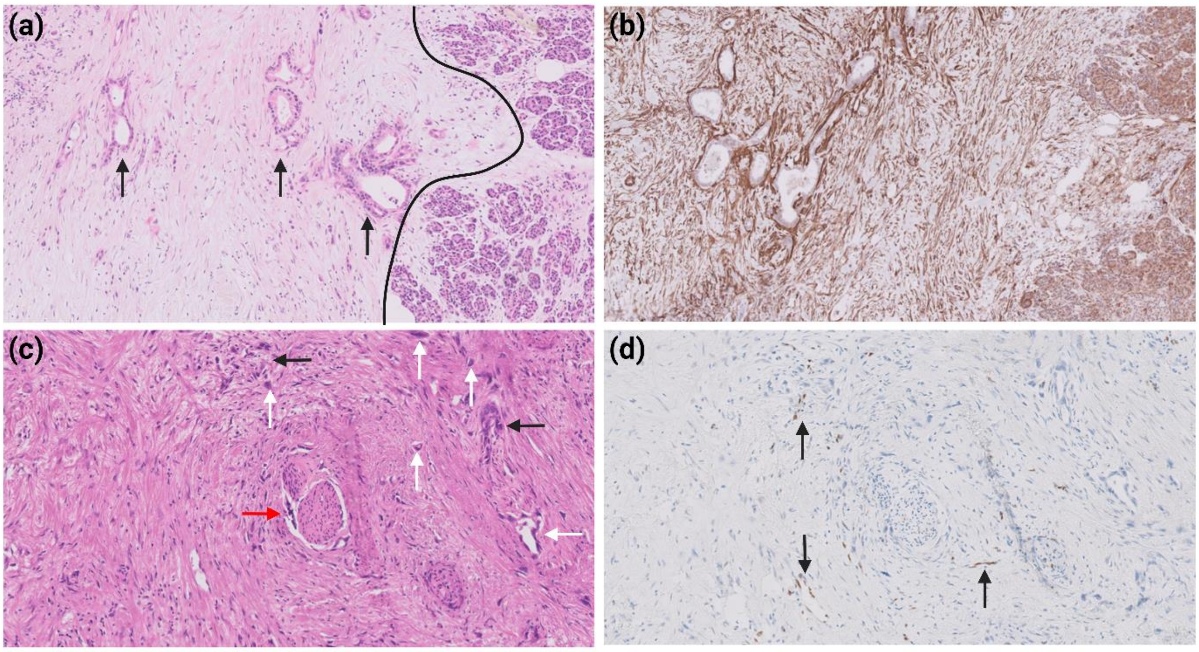

Supplementary Figure S11. H&E and immunohistochemical staining of representative PDAC samples illustrating typical characteristics of the PDAC. (a) H&E staining of a PDAC sample at the invasive front of the tumor (black line). Note the abundant amount of desmoplastic stroma surrounding neoplastic ductular structures around the invasive carcinoma (left, black arrows). (b) Immunohistochemical staining for alpha smooth muscle actin (α-SMA) of the same tumor as shown in (a), illustrating myofibroblastic differentiation of the tumor stroma. (c) Different tumor composed of perineural invasion (orange horizontal arrow), isolated tumor cells (white vertical arrows) and poorly formed ducts (black horizontal arrows) embedded in abundant desmoplastic stroma consisting of activated fibroblasts and myofibroblasts. (d) Immunohistochemical staining for ERG, a nuclear marker of endothelial cells (brown), of the same tumor as shown in (c), illustrating sparse vasculature consisting of thin-walled capillary-size vessels with scanty luminal diameter (black arrows).
